# Testing the Hypothesis of Multiple Origins of Holoparasitism in Orobanchaceae: Phylogenetic Evidence from the Last Two Unplaced Holoparasitic Genera, *Gleadovia* and *Phacellanthus*

**DOI:** 10.3389/fpls.2017.01380

**Published:** 2017-08-15

**Authors:** Weirui Fu, Xiaoqing Liu, Naixin Zhang, Zhiping Song, Wenju Zhang, Ji Yang, Yuguo Wang

**Affiliations:** Ministry of Education Key Laboratory for Biodiversity Science and Ecological Engineering, School of Life Sciences, Institute of Biodiversity Science, Fudan University Shanghai, China

**Keywords:** Orobanchaceae, *Gleadovia*, *Phacellanthus*, phylogenetic position, origins of holoparasitism

## Abstract

Orobanchaceae is the largest family among the parasitic angiosperms. It comprises non-parasites, hemi- and holoparasites, making this family an ideal test case for studying the evolution of parasitism. Previous phylogenetic analyses showed that holoparasitism had arisen at least three times from the hemiparasitic taxa in Orobanchaceae. Until now, however, not all known genera of Orobanchaceae were investigated in detail. Among them, the unknown phylogenetic positions of the holoparasites *Gleadovia* and *Phacellanthus* are the key to testing how many times holoparasitism evolved. Here, we provide clear evidence for the first time that they are members of the tribe Orobancheae, using sequence data from multiple loci (nuclear genes ITS, *PHYA, PHYB*, and plastid genes *rps2, mat*K). *Gleadovia* is an independent lineage whereas *Phacellanthus* should be merged into genus *Orobanche* section *Orobanche*. Our results unambiguously support the hypothesis that there are only three origins of holoparasitism in Orobanchaceae. Divergence dating reveals for the first time that the three origins of holoparasitism were not synchronous. Our findings suggest that holoparasitism can persist in specific clades for a long time and holoparasitism may evolve independently as an adaptation to certain hosts.

## Introduction

The family Orobanchaceae belongs to the eudicot order Lamiales. It comprises more than 2,000 species in 90 genera with lifestyles from fully autotrophic to completely heterotrophic, and thus becomes an ideal system to study the origin of holoparasitism (McNeal et al., 2013). Orobanchaceae was established first by Ventenat (1799). Initially, its members were defined as the plants which cannot photosynthesize. Subsequent researchers merged some hemiparasites in Scrophulariaceae into Orobanchaceae based on evidence from morphology and anatomy (von Wettstein, 1891; Bellini, 1907; Boeshore, 1920; Armstrong and Douglas, 1989). Following a phylogenetic analysis of the plastid gene *rps2*, the free-living genus *Lindenbergia* was regarded as a member of Orobanchaceae (Young et al., 1999). This proposal was also supported by sequence data of another plastid gene, *mat*K (Wolfe et al., 2005). In the latest classification by the Angiosperm Phylogeny Group (APG), 2016), the non-parasitic *Lindenbergia* and Rehmanniaceae were placed in Orobanchaceae. Phylogeny based on single plastid genes such as *mat*K, *rbcL*, and *rps2* partially resolved the phylogenetic relationships within Orobanchaceae (dePamphilis et al., 1997; Wolfe and dePamphilis, 1998; Young et al., 1999; Young and dePamphilis, 2005; Park et al., 2008), and nuclear genes (*PHYA* and ITS including the ITS1-5.8S-ITS2 region) provided improved resolution among species in Orobanchaceae (Wolfe et al., 2005; Bennett and Mathews, 2006). Compared with combined multiple genes, however, single genes do not always provide strong and clear resolution because of the dearth of informative sites, especially for closely related species (Zou et al., 2008; Niu et al., 2013; Yang et al., 2014). In a recent study, three nuclear genes (ITS, *PHYA*, and *PHYB*) and two plastid genes (*rps2* and *mat*K) were combined to reconstruct relationships within Orobanchaceae (McNeal et al., 2013). The backbone of the Orobanchaceae tree including six well-supported clades (Clade I–Clade VI) was determined. Previous studies suggested more than three independent origins of holoparasitism in Orobanchaceae (dePamphilis et al., 1997; Young et al., 1999), whereas the five-gene analysis showed that the holoparasitic species are only located in three clades (III, V, and VI) after excluding several species such as *Alectra orobanchoides, Striga gesnerioides, Striga hermonthica*, and *Tozzia alpina* based on the observation of life cycles in detail. Therefore, McNeal et al. (2013) concluded that holoparasitism had evolved independently three times in Orobanchaceae. Until now, however, this has not been tested on a sample of all holoparasitic species in Orobanchaceae. Furthermore, the order of origination time of the holoparasite lineages remains unknown.

The genera *Gleadovia* and *Phacellanthus* are just two ignored groups due to the lack of sampling for gene sequencing. They were placed into Orobanchaceae according to the number of valves, parietal placentas, and the shape of bracts and corolla (Zhang, 1990; Zhang and Tzvelev, 1998). *Gleadovia* comprises two species: *Gleadovia ruborum* is distributed in the northwest of the Himalaya, and *Gleadovia mupinensis* is endemic to southwest China. Both of them are small holoparasitic herbs. Our field observation showed that *G. mupinensis* parasitizes on species of *Rubus. Phacellanthus* is a monotypic genus which only contains a holoparasite, *Phacellanthus tubiflorus*. It is distributed in most provinces of China, as well as in Korea, Mongolia, and Russia. Although the originally recorded specimens parasitized on the roots of *Fraxinus* species, Xu et al. (1991) found that its host was *Tilia mandschurica* in Changbaishan nature reserve, China. In recent years, there were rarely reports about *Gleadovia* and *Phacellanthus* found in the wild (Chung et al., 2010; Liu et al., 2012). Ambiguous host information and uncertain time for collecting make it very difficult to resample *Gleadovia* and *Phacellanthus* from their type localities. Coupled with environmental changes and the impact of human activities, to collect the two holoparasitic genera successfully becomes a challenging job. Fortunately, after years of investigation, at last we sampled *G. mupinensis* and *P. tubiflorus* in the provinces Sichuan and Heilongjiang, China, respectively.

The phylogenetic positions of 12 holoparasitic genera were determined by McNeal et al. (2013), but those of some genera in Orobanchaceae including *Gleadovia* and *Phacellanthus* have still not been resolved by means of molecular methods. Moreover, *Eremitilla mexicana* was a newly reported holoparasitic species in Orobanchaceae (Yatskievych and Jiménez, 2009), which was suggested as a member of the clade including *Epifagus, Conopholis*, and *Boschniakia* based on ITS and *rps2* data (Mathews et al., 2008). McNeal et al. (2013) mentioned they hadn't sampled *Phelypaea*. In fact, Nicolson (1975) had proposed *Diphelypaea* as a replacement name for this genus. Phylogenetic analysis of ITS sequences plus karyotype evidence suggested that *Diphelypea* is sister to genus *Orobanche* section *Orobanche* (Schneeweiss et al., 2004a,b). Recently, ITS phylogeny provided clear evidence to merge the unplaced genus *Platypholis* into *Orobanche* (Li et al., 2017). Therefore, *Gleadovia* and *Phacellanthus* are the last two holoparasitic genera whose phylogenetic positions remain unclear. These two genera together with *Mannagettaea* and *Christisonia* had been placed in Gleadovieae rather than Orobancheae according to morphological characters such as type and position of inflorescence and absence/presence of mechanical tissue (Zhang and Tzvelev, 1998). But the latter two genera were proposed as members of Orobancheae and Buchnereae, respectively (McNeal et al., 2013). McNeal et al. (2013) did not accept Gleadovieae. Instead, they speculated that *Gleadovia* and *Phacellanthus* should belong to Orobancheae, although the samples of these two genera had not been obtained yet. Therefore, it is crucial to investigate the phylogenetic positions of *Gleadovia* and *Phacellanthus* for correctly understanding the origins of holoparasitism in Orobanchaceae. We reason that if they do not nest in the three holoparasitic clades, more than three origins of holoparasitism will be found; otherwise, three origins will be determined. In this study, we revisited the phylogeny of Orobanchaceae based on the combined sequence data of multiple DNA regions (ITS, *PHYA, PHYB, rps2*, and *mat*K) to identify the phylogenetic positions of *Gleadovia* and *Phacellanthus*. The number of origins of holoparasitism in Orobanchaceae was re-tested and the divergence dates were estimated to uncover the order of these origins.

## Materials and methods

### Plant materials and DNA extraction

We collected *G. mupinensis* and *P. tubiflorus* which represent two holoparasitic genera from the provinces Sichuan and Heilongjiang, China, respectively. Voucher specimens were deposited in the herbarium of Fudan University (FUS). Total genomic DNA was extracted from fresh tissues dried with silica-gel following the CTAB extraction method (Doyle and Doyle, 1987).

### PCR amplification and sequencing

Specific primers of five genes (Table S1) were used for polymerase chain reaction (PCR). Primers used to amplify the nuclear genes *PHYA* and *PHYB* as previously described (Bennett and Mathews, 2006; McNeal et al., 2013) did not work in *G. mupinensis* and *P. tubiflorus*; we designed new primers (Table S1) according to the sequences of closely related taxa inferred from phylogeny of plastid (*rps2* and *mat*K) data. Each reaction volume of 50 μl contained ~150 ng total DNA, 5 μl of 10X PCR buffer, 6 μl of MgCl_2_ (2.5 mmol·l^−1^), 8 μl of dNTP mixture (2.5 mmol·l^−1^), 3 μl of each primer (10 μmol·l^−1^), and 2.5 units of Red Taq DNA polymerase. The running programs for these genes are presented in Table S2. PCR products were visualized on 1% agarose gels and purified using Gel Extraction System B Kit (BioDev-Tech, Beijing, China) according to the manufacturer's instructions. The purified products were sequenced directly with BigDye Terminator 3.1 Cycle Sequencing Kit (Applied Biosystems) and run in ABI PRISM 377XL DNA Autosequencer. All sequences generated in this study were submitted to GenBank (accession numbers: KY706614–KY706632).

### Phylogenetic analyses

The sequences of five genes from *G. mupinensis* and *P. tubiflorus* were assembled by the software Seqman II 5.05 (DNAStar, London, UK). Sequence data of other species in Orobanchaceae were obtained from GenBank (Table S3). The alignment was undertaken by Clustal X (Larkin et al., 2007) and adjusted manually. All data sets were partitioned by gene, and gaps were treated as missing data. Modeltest 3.5 (Posada and Crandall, 1998) was executed to select the best fitting evolutionary model for each partition under the Akaike Information Criterion (AIC). Maximum likelihood (ML) and Bayesian inference (BI) analyses were performed on the independent and combined data sets for ITS, *PHYA, PHYB, rps2*, and *mat*K. ML analyses were run in RAxML 7.0.4 (Stamatakis, 2006); the general time reversible (GTR) model and gamma distribution were used for all partitions (McNeal et al., 2013) because it is impossible to specify different models to different partitions in RAxML. Support values for branches of the ML trees were assessed based on 1,000 bootstrap replicates. Before combining all data sets, the partition homogeneity test was used to check the congruence among the five-gene data sets in PAUP^*^ 4.0b10 (Swofford, 2002). Bayesian analyses were performed in MrBayes 3.1.2 (Huelsenbeck and Ronquist, 2001). Each partition was assigned its own nucleotide substitution model. We ran 25 million generations sampling every 1,000 generations. Stationarity of each analysis was assessed using Tracer 1.6.0 (Rambaut and Drummond, 2012) by checking the effective sample size (ESS) values. Completion was determined when the average standard deviation of split frequencies fell below 0.01. The 50% majority rule consensus trees were obtained after the first 25% of the samples were removed as burn-in.

In order to compare the resolution of different partitioning schemes, we also divided the data by codon to construct the phylogeny of Orobanchaceae based on single and combined gene data, respectively. Each protein-coding gene (*PHYA, PHYB, rps2, mat*K) was partitioned by codon (1st, 2nd, and 3rd codon position) and the best fitting models were selected by Modeltest 3.5. The concatenated five-gene data set was partitioned into 13 blocks, including one for the ITS region and 12 for the 1st, 2nd, and 3rd codon positions of each protein-coding gene. ML analyses were conducted with RAxML 7.0.4 under the GTR + G model and Bayesian inference was run in MrBayes 3.1.2 with different nucleotide substitution models for each partition.

### The timing of origin of holoparasitic clades in orobanchaceae

Bayesian Evolutionary Analysis by Sampling Trees (BEAST 1.8.2; Drummond et al., 2012) was used to estimate the divergence time in Orobanchaceae. Bayesian Evolutionary Analysis Utility (BEAUti 1.8.2) was used to output BEAST input files. Owning to the lack of a reliable fossil record within the family (Soltis et al., 2011; Tank et al., 2015; Uribe-Convers and Tank, 2015), two external fossil calibration points (Call and Dilcher, 1992; Collinson et al., 1993) were used in the dating analysis. Both the stem age of Solanaceae and the age of the most recent common ancestor of *Pedicularis* and *Olea* were constrained with the same lognormal distribution prior (an offset of 44.3 Mya, a mean of 1.5, and a standard deviation of 0.5; Zanne et al., 2014). These two nodes are relatively close to Orobanchaceae (Zanne et al., 2014). Ten independent Monte chains Carlo Markov (MCMC) were conducted to ensure convergence in divergence time. Each run consisted of 25 million generations (sampling every 1,000 steps) with the GTR + G model, a Yule tree prior and an uncorrelated lognormal clock. Tracer 1.6.0 (Rambaut and Drummond, 2012) and an R package, RWTY (Warren et al., 2017), were used to assess convergence and stationarity of each MCMC chain. The RWTY test showed that each MCMC chain reached convergence within 5 million generations. Therefore, our analyses of 25 million generations guarantee convergence and stationarity of each MCMC chain. The samples from each run were combined by LogCombiner 1.8.2, until ESS ≥ 200. The final maximum clade credibility tree was generated using TreeAnnotator 1.8.2 after removing 10% of the samples as burn-in. The topology and node height with 95% highest posterior density (HPD) were visualized in FigTree 1.3.1 (Rambaut, 2006).

## Results

**Figure 1:**
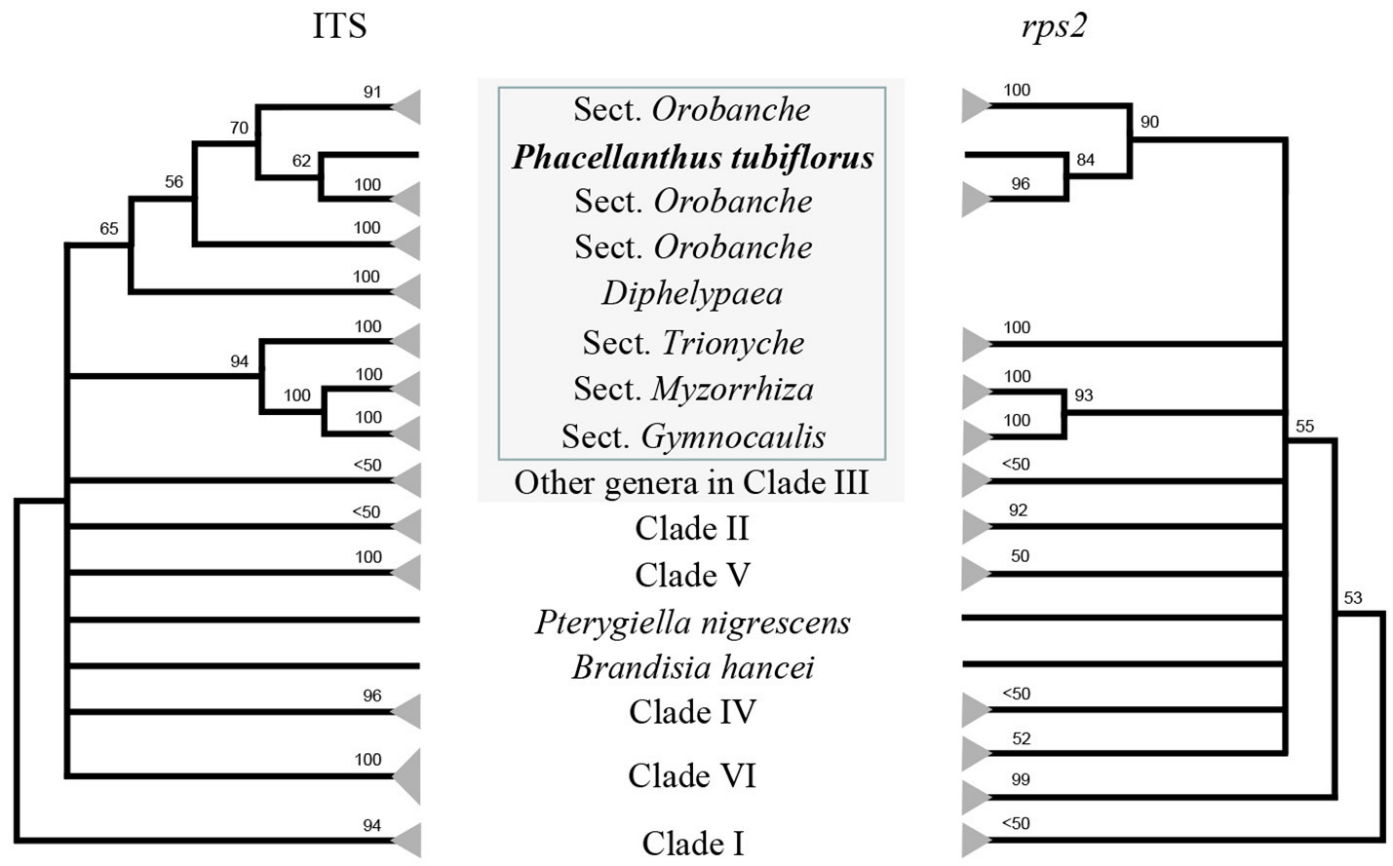
Schematic maximum likelihood phylogenetic trees based on the sequences of ITS **(left)** and *rps2* **(right)** showing the evidence that *Phacellanthus tubiflorus* is nested in the section *Orobanche* of the genus *Orobanche*. Numbers above branches are bootstrap values, only bootstrap values >50 are shown. Taxa in gray shadow represent Clade III = Orobancheae. Four sections of *Orobanche* and *Diphelypaea* are indicated in the box. The expanded trees are presented in Figures S7–S10.

As expected, our ML trees do not present any difference with those of McNeal et al. (2013) in topology among the six main clades of Orobanchaceae except some details. The combined five-gene data provides a clearer resolution than any single gene. All phylogenetic trees show that *P. tubiflorus* is clustered with the members of the genus *Orobanche* within Clade III, i.e., Orobancheae (Figure 1, Figures S1–S11). Except for the trees based on ITS or *rps2* sequences which present poor resolution in Clade III, all trees show *G. mupinensis* nested in Orobancheae (Figures S1–S11).

ML analyses based on different partitioning schemes (by gene and by codon) obtained similar results with slightly different support values. The results of the BI analyses are consistent with those of the ML analyses concerning the main clades and the phylogenetic positions of *P. tubiflorus* and *G. mupinensis*, expect for the *PHYA* tree which shows poor resolution based on codon partition. The phylogenetic information from ML and BI trees of combined five-gene data based on two kinds of partitioning schemes (Figures S11–S14) is summarized in Table 1. The corresponding ML results from single genes and combined plastid genes are attached in supplementary materials (Tables S4, S5).

**Table 1:** Summarized results of phylogenetic analyses in Orobanchaceae based on the combined five-gene data.

	**Maximum likelihood**		**Bayesian inferences**	
**Method and partitioning scheme**	**By gene**	**By codon**	**By gene**	**By codon**
Species	127	127	127	127
Nucleotides after alignment	6,659	6,851	6,659	6,851
Parsimony-informative characters	3,339	3,400	3,339	3,400
Parasitic taxa form a clade	96%	94%	1.00	0.98
Clade I exists	100%	100%	1.00	0.99
Clade II exists	100%	100%	1.00	0.99
Holoparasitic Clade III exists	100%	100%	1.00	0.97
*Gleadovia* is an independent lineage	75%	77%	0.97	0.95
*Orobanche* forms a clade	95%	96%	1.00	0.97
*Phacellanthus tubiflorus* clusters with the species of section *Orobanche*	100%	100%	1.00	0.99
Clade IV exists	100%	100%	1.00	0.98
Clade V exists	100%	100%	1.00	0.98
The species of holoparasitic *Lathraea* form a clade	100%	100%	1.00	1.00
Clade VI exists	100%	100%	1.00	0.96
Holoparasitic species in Clade VI form a clade	99%	100%	1.00	0.99
*Brandisia hancei* is an independent lineage	69%	64%	1.00	0.96

*Bootstrap values and posterior probabilities are indicated in each analysis. All clades showed here follows McNeal et al. (2013): Clade I, Lindenbergia; Clade II, Cymbarieae; Clade III, Orobancheae; Clade IV, Pedicularideae; Clade V, Rhinantheae except Pterygiella nigrescens; Clade VI, Buchnereae*.

Our divergence dating analyses clearly show that the three holoparasitic clades in Orobanchaceae originated non-synchronously (**Figure 3**, Figure S15). The first origin of holoparasitism was found in Clade III, followed by the clade (*Hyobanche*, (*Harveya*, (*Aeginetia, Christisonia*))) within Clade VI and the genus *Lathraea* in Clade V.

## Discussion

### Gene selection for phylogenetic reconstruction

**Figure 2:**
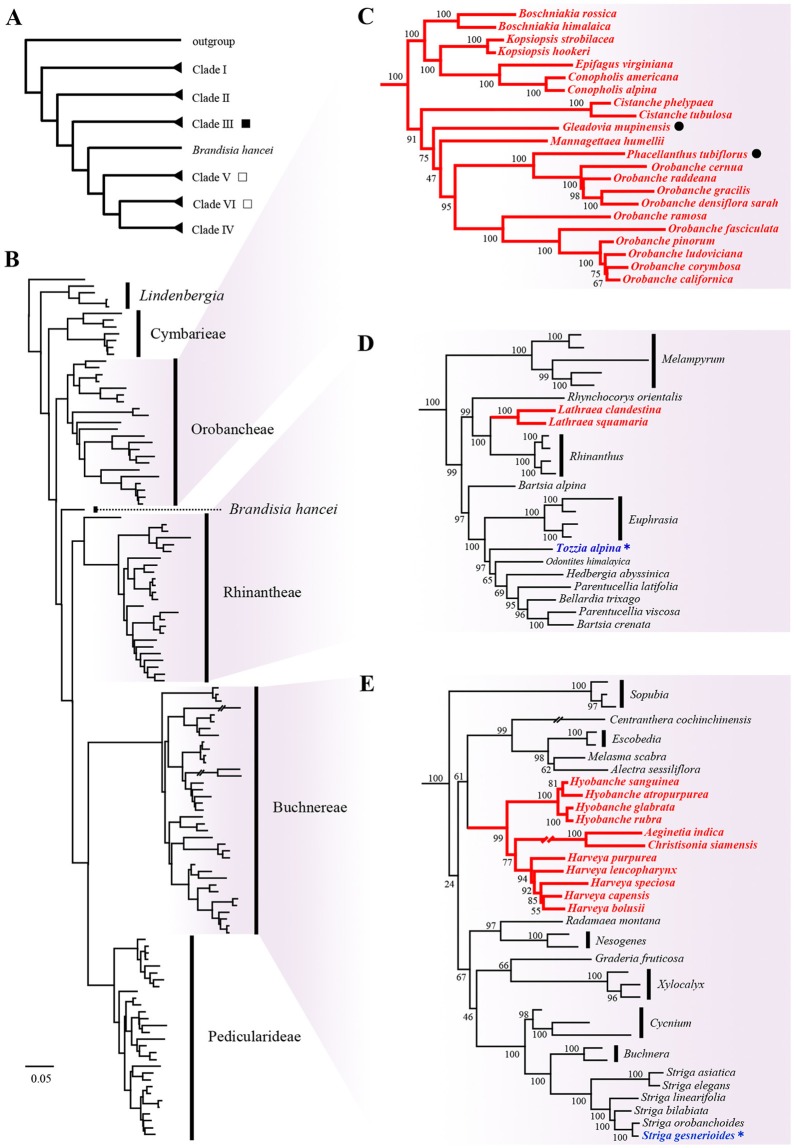
Maximum likelihood phylogenetic trees of Orobanchaceae inferred from five combined genes. Simplified major backbone of Orobanchaceae shows the relationships among the main clades **(A)** and among corresponding taxa **(B)**. The name of each clade is indicated. □ means there are holoparasites in the clade, ■ means this clade contains holoparasites only. Details including bootstrap values and GenBank accession numbers are presented in Figure S11 and Table S3. Trees are rooted with *Paulownia tomentosa*. Three clades containing holoparasitic taxa are presented on the right: Clade III, Orobancheae **(C)**; Clade V, Rhinantheae **(D)**; Clade VI, Buchnereae **(E)**. Numbers above the branches indicate the maximum likelihood bootstrap values resulting from 1,000 replicates. Holoparasitic taxa are highlighted in red bold font. The taxa which were incorrectly placed in holoparasitic taxa in previous work are indicated in blue bold font and marked with an asterisk (^*^). The phylogenetic positions of *Gleadovia* and *Phacellanthus* are marked with • in Clade III, Orobancheae **(C)**.

Of the genes used in our ML analyses, the nuclear gene ITS has a limited capacity to resolve the phylogeny of Orobanchaceae at the genus level (Schneeweiss et al., 2004a; Wolfe et al., 2005; Gussarova et al., 2008). Our ML tree based on ITS data shows good support in four clades (I, IV, V, and VI), but poor support for Clade II [maximum likelihood bootstrap (MLBS) < 50%]. Although Clade III collapses in the ML tree, section *Orobanche* forms a clade, in which the relationships among most species obtain strong support (Figure S1). The plastid gene *rps2*, which is known to be retained in all hemi- and holoparasites in Orobanchaceae, behaves similarly to ITS. The coding gene *mat*K has been widely used to infer phylogenetic relationships among closely related groups. Compared with *rps2*, combined plastid data (*rps2* + *mat*K) can resolve the basal clades and support the relationships of the species within the main clades well. The nuclear genes *PHYA* and *PHYB* are associated with seed germination (Mathews and Donoghue, 2000; Franklin and Whitelam, 2005). Previous studies suggested that *PHYA* is the most useful single locus to resolve the phylogenetic relationships within Orobanchaceae (Mathews and Donoghue, 2000; Bennett and Mathews, 2006), but the phylogenetic trees of *PHYB*, by contrast, showed higher resolution of the relationships among and within the main clades (Figures S5, S6, Table S4). In general, phylogenetic resolution of the combined data is superior to that of any single gene. To gain a better understanding of the phylogenetic relationships among the species of Orobanchaceae, it is necessary to combine nuclear genes with plastid genes as in the previous study by McNeal et al. (2013). In this study, our phylogenetic analyses based on the combined data show strong support among and within the major clades in Orobanchaceae (Figure 2).

### Phylogenetic position of *Phacellanthus* and *Gleadovia*

All ML trees based on single or combined genes support that *P. tubiflorus* clusters with the members of the Orobancheae (Figures 1, 2, Figures S1–S11). Traditionally, the genus *Orobanche* was divided into four sections: *Gymnocaulis, Myzorrhiza, Trionychon*, and *Orobanche*. As far as *Orobanche* in Clade III is concerned, our phylogenetic analyses based on both single and combined genes obtained the same results as the previous study by Schneeweiss et al. (2004a), i.e., *Orobanche* falls into two lineages: the subgenus *Orobanche* which contains the sections *Orobanche* and *Diphelypaea*, and the subgenus *Phelipanche* which contains the sections *Gymnocaulis, Myzorrhiza*, and *Trionychon*. Almost all ML trees in this study strongly support *P. tubiflorus* as the closest relative of section *Orobanche* (99 or 100% MLBS), with the exception of the ITS tree (72% MLBS). Independent analysis of either ITS or *rps2* supports that *P. tubiflorus* is nested in section *Orobanche* (Figure 1, Figures S7–S10). Although we looked for morphological differences between *Phacellanthus* and the section *Orobanche*, we found only one: the dehiscing capsule has three valves in *Phacellanthus* but two valves in the section *Orobanche*. Except for this difference, they present high similarities in the shape of the stem, leaf, calyx, corolla, and capsule, the arrangement of the leaves, the number of stamina, and so on. Considering all the evidence above, it is better to merge *Phacellanthus* into section *Orobanche*.

Unlike the situation of *Phacellanthus*, almost all analyses from single and combined genes support *G. mupinensis* as an independent lineage. Even if ITS shows poor resolution in Orobancheae, *G. mupinensis* forms a polytomy with several large clades (Figure S1, Table S4). ML trees constructed with *PHYA, PHYB*, two plastid genes or combined data from five genes (Figures S5, S6, Table S4) group *G. mupinensis* within Clade III, near to *Orobanche*, but *G. mupinensis* is not nested in the clade including *Boschniakia, Epifagus*, and *Conopholis*. The phylogenetic positions of *Gleadovia* and *Phacellanthus* clearly support the tribe Orobancheae revised by McNeal et al. (2013), but do not support the tribe Gleadovieae which groups *Gleadovia* with the genera *Mannagettaea, Phacellanthus*, and *Christisonia* (Zhang and Tzvelev, 1998). Indeed, only one chamber is fertile in *Christisonia* and *Aeginetia*, supporting their sister-group relationship in Clade VI. However, the anthers of the species of *Gleadovia* and the members of tribe Orobancheae are fertile and have two equal chambers. By contrast, *Gleadovia* shares more morphological similarities with *Mannagettaea* and *Phacellanthus* despite the slight differences in the number of placentae among these three genera and the number of carpels between *Gleadovia* and *Phacellanthus*.

### Origins of holoparasitism

Several species in Orobanchaceae such as *A. orobanchoides, S. gesnerioides, S. hermonthica*, and *T. alpina* were regarded as holoparasites before McNeal et al. (2013) showed that these species retain functional chlorophyll and photosynthesize at least in part of their life cycle, although sometimes the photosynthesis rates are very low. Without considering these species, our results support three origins of holoparasitism in Orobanchaceae. We note that certain hemiparasitic genera cannot be included in this study due to the limitation of sampling. Because Clade III is strictly restricted at the circumscription of Orobanchaceae *sensu stricto* containing only holoparasites, and the holoparasites in Clade V consist of only the species of *Lathraea*, the holoparasitic clade (*Hyobanche*, (*Harveya*, (*Aeginetia, Christisonia*))) within Clade VI becomes the most likely lineage in which hemiparasitic species might be nested. However, these four closely related holoparasitic genera are morphologically so similar that their holoparasitism is most likely homologous. Any hemiparasites nested among them would therefore have regained the ability to photosynthesize. We consider this highly unlikely because no such reversal from holo- to hemiparasitism has been reported to date; but even in that case, the number of origins of holoparasitism would remain unchanged. Thus, if we accept that *A. orobanchoides, S. gesnerioides, S. hermonthica*, and *T. alpina* are hemiparasites (McNeal et al., 2013) and that *Eremitilla* and *Platypholis* belong to Clade III, there were three independent origins of holoparasitism in Orobanchaceae, each time from hemiparasites.

**Figure 3:**
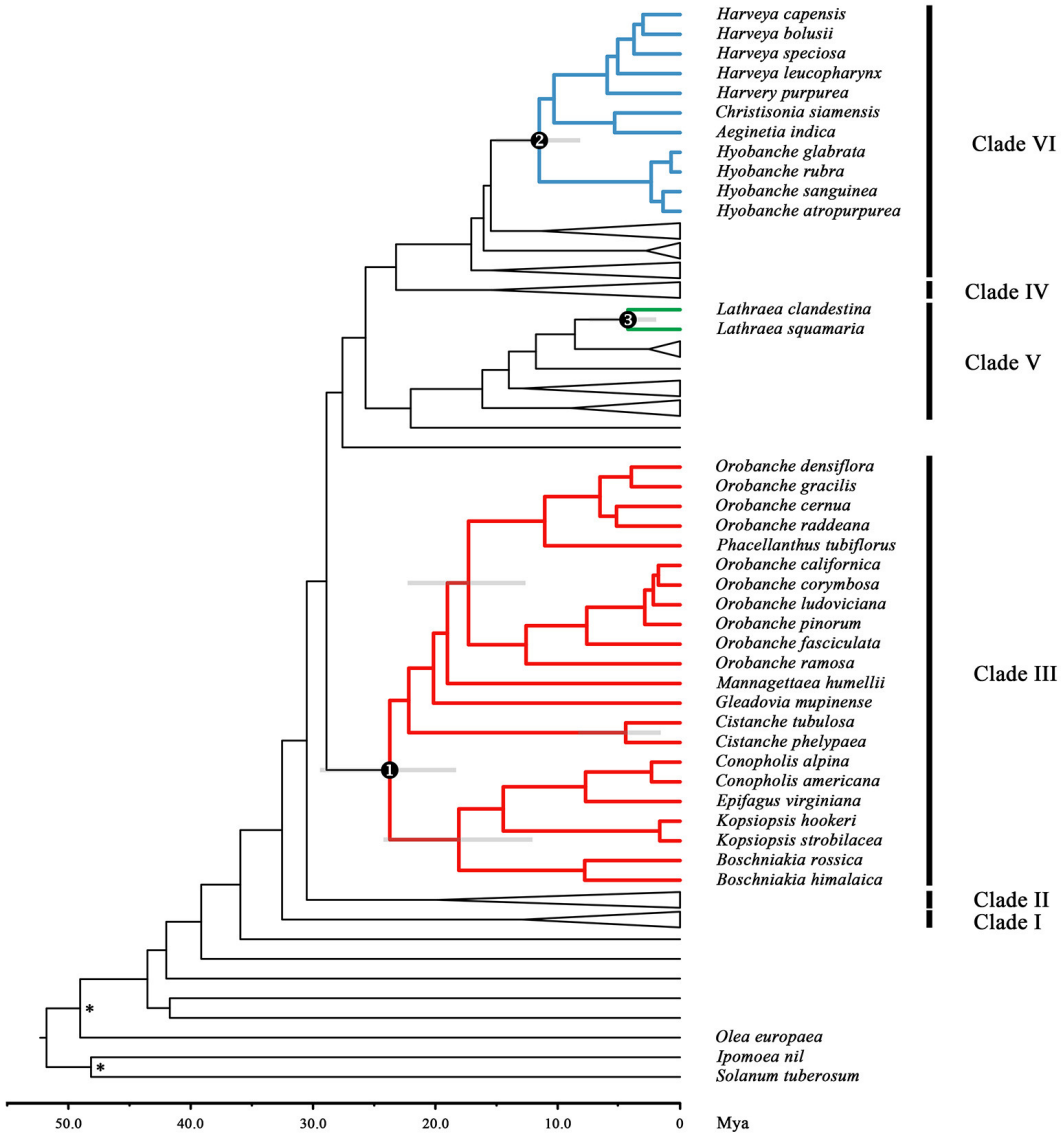
The divergence times of the main clades in Orobanchaceae showing three non-synchronous origins of the holoparasitic clades. Topology of 134 species in Orobanchaceae and seven outgroup species, *Paulownia tomentosa, Sesamum indicum, Scrophularia arguta, Antirrhinum majus, Olea europaea, Ipomoea nil*, and *Solanum tuberosum*, obtained from a combined analysis of a 6,659 bp alignment of plastid and nuclear DNA sequence data. Calibration nodes are marked with an asterisk (^*^). Numbered circles and different colors mark the crown groups of the three holoparasitic clades in Orobanchaceae. The gray bars are the 95% HPD intervals for the divergence time estimates. Time in millions of years ago (Mya) is represented by the scale axis below the tree. Major clades referred to in the text are indicated: Clade I, *Lindenbergia*; Clade II, Cymbarieae; Clade III, Orobancheae; Clade IV, Pedicularideae; Clade V, Rhinantheae (except *Pterygiella nigrescens*), and Clade VI, Buchnereae. The expanded tree is presented in Figure S15.

Orobanchaceae was estimated to have a mid-Tertiary Laurasian origin (Wolfe et al., 2005; Soltis et al., 2011; Tank et al., 2015; Uribe-Convers and Tank, 2015). Based on the fossil record of close relatives of Orobanchaceae, we can compare the relative divergence times of the holoparasitic clades (Figure 3, Figure S15). Our data show that the oldest holoparasitic clade is Orobancheae. The second holoparasitic clade is (*Hyobanche*, (*Harveya*, (*Aeginetia, Christisonia*))) in Buchnereae. The crown group of this clade seems to postdate those of *Orobanche* and the clade (*Boschniakia*, (*Kopsiopsis*, (*Epifagus, Conopholis*))). The youngest holoparasitic clade comprises only the genus *Lathraea*. The age of this genus is almost equal to the divergence between *Cistanche phelypaea* and *C. tubulosa*.

## Conclusion and outlook

Our phylogenetic analyses based on multiple loci demonstrate that there are only three origins of holoparasitism in Orobanchaceae. Both *Gleadovia* and *Phacellanthus* fall into Orobancheae. *Gleadovia* is an independent lineage whereas *Phacellanthus* should be merged into *Orobanche* section *Orobanche*. We show for the first time that the three origins of holoparasitism happened at three different times. Further, we note that different holoparasitic species in Orobanchaceae, even when they belong to the same genus, have hosts from distinct genera, implying that the appearances of holoparasitism in different evolutionary periods bear on the intimate interactions with their own hosts. Our findings suggest that holoparasitism can persist in specific clades for a long time and holoparasitism may evolve repeatedly as an adaptation to different hosts. Besides further study of the hemiparasitic lineages, special attention should be paid directly in the future to the parallel evolution of holoparasitism, the effects of host shifts on the speciation of parasites, and the mechanisms underlying coevolution between parasites and their hosts.

## Author contributions

WF collected plant materials, performed the experiments, analyzed data, and wrote the first version of the manuscript. XL and NZ revised the manuscript, analyzed the results. ZS, WZ, and JY analyzed the results and revised the manuscript. YW designed experiments, supervised the study, co-wrote and revised the manuscript. All authors contributed to and approved the final manuscript.

### Conflict of interest statement

The authors declare that the research was conducted in the absence of any commercial or financial relationships that could be construed as a potential conflict of interest.

## Supplementary material

Figure S1 Phylogenetic tree of Orobanchaceae including *Gleadovia mupinensis* and *Phacellanthus tubiflorus* based on ITS data with ML method.

Figure S2 Phylogenetic tree of Orobanchaceae including *Gleadovia mupinensis* and *Phacellanthus tubiflorus* based on *rps2* data with ML method.

Figure S3 Phylogenetic tree of Orobanchaceae including *Gleadovia mupinensis* and *Phacellanthus tubiflorus* based on *mat*K data with ML method.

Figure S4 Phylogenetic tree of Orobanchaceae including *Gleadovia mupinensis* and *Phacellanthus tubiflorus* based on *rps2* + *mat*K data with ML method.

Figure S5 Phylogenetic tree of Orobanchaceae including *Gleadovia mupinensis* and *Phacellanthus tubiflorus* based on *PHYA* data with ML method.

Figure S6 Phylogenetic tree of Orobanchaceae including *Gleadovia mupinensis* and *Phacellanthus tubiflorus* based on *PHYB* data with ML method.

Figure S7 Expanded phylogenetic trees based on ITS sequences for *Orobanche* with ML method.

Figure S8 Expanded phylogenetic trees based on ITS sequences for Orobanchaceae with ML method.

Figure S9 Expanded phylogenetic trees based on *rps2* sequences for *Orobanche* with ML method.

Figure S10 Expanded phylogenetic trees based on *rps2* sequences for Orobanchaceae with ML method.

Figure S11 Expanded maximum likelihood phylogenetic tree of Orobanchaceae inferred from the combined five-gene data set (*PHYA, PHYB*, ITS, *mat*K, and *rps2*) partitioned by gene.

Figure S12 Maximum likelihood phylogenetic tree of Orobanchaceae inferred from the combined five-gene data set (*PHYA, PHYB*, ITS, *mat*K, and *rps2*) partitioned by codon.

Figure S13 Bayesian phylogenetic tree of Orobanchaceae inferred from the combined five-gene data set (*PHYA, PHYB*, ITS, *mat*K, and *rps2*) partitioned by gene.

Figure S14 Bayesian phylogenetic tree of Orobanchaceae inferred from the combined five-gene data set (*PHYA, PHYB*, ITS, *mat*K, and *rps2*) partitioned by codon.

Figure S15 Divergence time estimation of Orobanchaceae using combined five-gene data obtained from BEAST analysis.

Table S1 The primer information for PCR amplification and cycle sequencing.

Table S2 PCR amplification programs of each gene used in this study.

Table S3 GenBank accession numbers for species used in this study.

Table S4 Summarized results of maximum likelihood analyses in Orobanchaceae based on single gene and combined *mat*K and *rps2* (plastid) data partitioned by gene.

Table S5 Summarized results of maximum likelihood analyses in Orobanchaceae based on single gene and combined *mat*K and *rps2* (plastid) data partitioned by codon.

## References

[B1] Angiosperm Phylogeny Group (APG) (2016). An update of the Angiosperm Phylogeny Group classification for the orders and families of flowering plants: APG IV. Bot. J. Linn. Soc. 181, 1–20. 10.1111/boj.12385

[B2] Armstrong J. E. Douglas A. W. (1989). The ontogenetic basis for corolla aestivation in Scrophulariaceae. Bull. Torrey Bot. Club 116, 378–389. 10.2307/2996628

[B3] Bellini R. (1907). Criteri per una nuova classificazione delle Personatae (Scrophulariaceae et Rhinathaceae). *Ann*. Bot. 6, 131–145.

[B4] Bennett J. R. Mathews S. (2006). Phylogeny of the parasitic plant family Orobanchaceae inferred from phytochrome A. Amer. J. Bot. 93, 1039–1051. 10.3732/ajb.93.7.1039 21642169

[B5] Boeshore I. (1920). The morphological continuity of Scrophulariaceae and Orobanchaceae. Contr. Bot. Lab. Univ. Penn. 5, 139–177.

[B6] Call V. B. Dilcher D. L. (1992). Investigations of angiosperms from the Eocene of southwestern North America: samaras of *Fraxinus wilcoxiana* berry. Rev. Palaeobot. Palynol. 74, 249–266. 10.1016/0034-6667(92)90010-E

[B7] Chung S. W. Hsu T. C. Peng C. I. (2010). *Phacellanthus* (Orobanchaceae), a newly recorded genus in Taiwan. Bot. Stud. 51, 531–536. Available online at: http://ejournal.sinica.edu.tw/bbas/content/2010/4/Bot514-13/Bot514-13.html

[B8] Collinson M. E. Boulter M. C. Holmes P. L. (1993). Magnoliophyta (‘Angiosperme’), in The Fossil Record 2, ed Benton M. J. (London: Chapman and Hall Press), 809–841.

[B9] dePamphilis C. W. Young N. D. Wolfe A. D. (1997). Evolution of plastid gene *rps2* in a lineage of hemiparasitic and holoparasitic plants: many losses of photosynthesis and complex patterns of rate variation. Proc. Natl. Acad. Sci. U.S.A. 94, 7367–7372. 10.1073/pnas.94.14.7367 9207097 PMC23827

[B10] Doyle J. J. Doyle J. L. (1987). A rapid DNA isolation for small quantities of fresh tissue. *Phytochem*. Bull. 19, 11–15.

[B11] Drummond A. J. Suchard M. A. Xie D. Rambaut A. (2012). Bayesian phylogenetics with BEAUti and the BEAST 1.7. Mol. Biol. Evol. 29, 1969–1973. 10.1093/molbev/mss075 22367748 PMC3408070

[B12] Franklin K. A. Whitelam G. C. (2005). Phytochromes and shade-avoidance responses in plants. Ann. Bot. 96, 169–175. 10.1093/aob/mci165 15894550 PMC4246865

[B13] Gussarova G. Popp M. Vitek E. Brochmann C. (2008). Molecular phylogeny and biogeography of the bipolar *Euphrasia* (Orobanchaceae): recent radiations in an old genus. Mol. Phylogenet. Evol. 48, 444–460. 10.1016/j.ympev.2008.05.002 18555702

[B14] Huelsenbeck J. P. Ronquist F. (2001). MRBAYES: Bayesian inference of phylogenetic trees. Bioinformatics 17, 754–755. 10.1093/bioinformatics/17.8.754 11524383

[B15] Larkin M. A. Blackshields G. Brown N. P. Chenna R. McGettigan P. A. McWilliam H.. (2007). Clustal W and Clustal X version 2.0. Bioinformatics 23, 2947–2948. 10.1093/bioinformatics/btm404 17846036

[B16] Li X. Jang T. S. Temsch E. M. Kato H. Takayama K. Schneeweiss G. M. (2017). Molecular and karyological data confirm that the enigmatic genus *Platypholis* from Bonin-islands (SE Japan) is phylogenetically nested within Orobanche (Orobanchaceae). J. Plant Res. 2, 273–280. 10.1007/s10265-016-0888-y PMC5318490 28004281

[B17] Liu P. Du C. Lu Y. Jiang Z. (2012). Three newly recorded genera of plant from Qinling Mountains. Acta Bot. Boreal Occid. Sin. 32, 1910–1912. 10.3969/j.issn.1000-4025.2012.09.028

[B18] Mathews S. Donoghue M. J. (2000). Basal angiosperm phylogeny inferred from duplicate phytochromes A and C. Inter. J. Plant Sci. 161, S41–S55. 10.1086/317582 10542147

[B19] Mathews S. Schneeweiss G. M. Yatskievych G. (2008). Phylogenetic affinities of a new holoparasitic genus of Orobanchaceae endemic to Guerrero, Mexico, inferred from molecular data, in Abstracts of Botany 2008 Conference (Vancouver). Available online at: http://2008.botanyconference.org/engine/search/index.php?func=detail&aid=858

[B20] McNeal J. R. Bennett J. R. Wolfe A. D. Mathews A. S. (2013). Phylogeny and origins of holoparasitism in Orobanchaceae. Amer. J. Bot. 100, 971–983. 10.3732/ajb.1200448 23608647

[B21] Nicolson H. D. (1975). *Diphelypaea (Orobanchaeae), nom. nov*. and other cauterizations on a nomenclatural hydra. Taxon 24, 651–657. 10.2307/1220740

[B22] Niu S. H. Li Z. X. Yuan H. W. Chen X. Y. Li Y. Li W. (2013). Transcriptome characterisation of *Pinus tabuliformis* and evolution of genes in the *Pinus* phylogeny. BMC Genomics 14:263. 10.1186/1471-2164-14-263 23597112 PMC3640921

[B23] Park J. M. Manen J. F. Colwell A. E. Schneeweiss G. M. (2008). A plastid gene phylogeny of the non-photosynthetic parasitic *Orobanche* (Orobanchaceae) and related genera. J. Plant Res. 121, 365–376. 10.1007/s10265-008-0169-5 18483784

[B24] Posada D. Crandall K. A. (1998). MODELTEST: testing the model of DNA substitution. Bioinformatics 14, 817–818. 10.1093/bioinformatics/14.9.817 9918953

[B25] Rambaut A. (2006). FigTree. Institute of Evolutionary Biology, University of Edinburgh, Edinburgh Available online at: http://tree.bio.ed.ac.uk/software/figtree/

[B26] Rambaut A. Drummond A. J. (2012). Tracer v1.6.0. Available online at: http://beast.bio.ed.ac.uk/Tracer

[B27] Schneeweiss G. M. Colwell A. Park J. M. Jang C. G. Stuessy T. F. (2004a). Phylogeny of holoparasitic *Orobanche* (Orobanchaceae) inferred from nuclear ITS sequences. Mol. Phylogenet. Evol. 30, 465–478. 10.1007/s10265-008-0169-5 14715236

[B28] Schneeweiss G. M. Palomeque T. Alison E. Colwell A. E. Schneeweiss H. W. (2004b). Chromosome numbers and karyotype evolution in holoparasitic *Orobanche* (Orobanchaceae) and related genera. Amer. J. Bot. 9, 439–448. 10.3732/ajb.91.3.439 21653400

[B29] Soltis D. E. Smith S. A. Cellinese N. Wurdack K. J. Tank D. C. Brockington S. F.. (2011). Angiosperm phylogeny: 17 genes, 640 taxa. Amer. J. Bot. 98, 704–730. 10.3732/ajb.1000404 21613169

[B30] Stamatakis A. (2006). RAxML-VI-HPC: maximum likelihood-based phylogenetic analyses with thousands of taxa and mixed models. Bioinformatics 22, 2688–2690. 10.1093/bioinformatics/btl446 16928733

[B31] Swofford D. L. (2002). PAUP^*^. Phylogenetic Analysis Using Parsimony (^*^and Other Methods). Version 4b10. Sunderland, MA: Sinauer Associates.

[B32] Tank D. C. Eastman J. M. Pennell M. W. Soltis P. S. Soltis D. E. Hinchliff C. E.. (2015). Nested radiations and the pulse of angiosperm diversification: increased diversification rates often follow whole genome duplications. New Phytol. 207, 454–467. 10.1111/nph.13491 26053261

[B33] Uribe-Convers S. Tank D. C. (2015). Shifts in diversification rates linked to biogeographic movement into new areas: an example of a recent radiation in the Andes. Amer. J. Bot. 102, 1854–1869. 10.3732/ajb.1500229 26542843

[B34] Ventenat E. P. (1799). ‘Orobanchoideae, in Tableau du Règne vegetal selon la Méthode de Jussieu, Vol. 2, ed Ventenat E. P. (Paris: J. Drisonnier Press), 292.

[B35] von Wettstein R. (1891). Scrophulariaceae, in Die Natürlichen Pflanzenfamilien, eds Engler A. Prantle K. (Leipzig: Engelmann Press), 39–107.

[B36] Warren D. L. Geneva A. J. Lanfear R. (2017). RWTY (R We There Yet): an R package for examining convergence of Bayesian phylogenetic analyses. Mol. Biol. Evol. 34, 1016–1020. 10.1093/molbev/msw279 28087773

[B37] Wolfe A. D. dePamphilis C. W. (1998). The effect of relaxed functional constraints on photosynthetic gene *rbcL* in photosynthetic and nonphotosynthetic parasitic plants. Mol. Biol. Evol. 15, 1243–1258. 10.1093/oxfordjournals.molbev.a025853 9787431

[B38] Wolfe A. D. Randle C. P. Liu L. Steiner K. E. (2005). Phylogeny and biogeography of Orobanchaceae. Folia Geobot. 40, 115–134. 10.1007/BF02803229

[B39] Xu G. Zhang X. Yin L. Bai Y. (1991). Phacellanthus tubiflorus. Plants 1, 48–49.

[B40] Yang Y. C. Yang S. H. Fang L. Li J. F. Zhong C. R. Zhou R. C. (2014). Phylogenetic position of *Sonneratia griffithii* based on sequences of the nuclear ribosomal internal transcribed spacer and 13 nuclear genes. J. Syst. Evol. 53, 47–52. 10.1111/jse.12102

[B41] Yatskievych G. Jiménez J. L. C. (2009). A new genus of holoparasitic Orobanchaceae from Mexico. Novon 19, 266–276. 10.3417/2008088

[B42] Young N. D. dePamphilis C. W. (2005). Rate variation in parasitic plants: correlated and uncorrelated patterns among plastid genes of different function. BMC Evol. Biol. 5:16. 10.1186/1471-2148-5-16 15713237 PMC554776

[B43] Young N. D. Steiner K. E. dePamphilis C. W. (1999). The evolution of parasitism in Scrophulariaceae/Orobanchaceae: plastid gene sequences refute an evolutionary transition series. Ann. Mo. Bot. Gard. 86, 876–893. 10.2307/2666173

[B44] Zanne A. E. Tank D. C. Cornwell W. K. Eastman J. M. Smith S. A. FitzJohn R. G.. (2014). Three keys to the radiation of angiosperms into freezing environments. Nature 506, 89–92. 10.1038/nature12872 24362564

[B45] Zhang Z. (1990). Orobanchaceae, in Fl. Reipubl. Popularis Sin., ed Wang W. (Beijing: Science Press), 69–124.

[B46] Zhang Z. Tzvelev N. N. (1998). Orobanchaceae, in Flora of China, eds Wu Z. Raven P. H. (Beijing: Science Press and Saint Louis; Missouri Botanical Garden Press), 229–243.

[B47] Zou X. H. Zhang F. M. Zhang J. G. Zang L. L. Tang L. Wang J. (2008). Analyses of 142 genes resolves the rapid diversification of the rice genus. Genome Biol. 9:R49 10.1186/gb-2008-9-3-r49 18315873 PMC2397501

