# Supplementary figures and images for: Testing the Hypothesis of Multiple Origins of Holoparasitism in Orobanchaceae: Phylogenetic Evidence from the Last Two Unplaced Holoparasitic Genera, Gleadovia and Phacellanthus
Source: Front Plant Sci. 2017 Aug 15;8:1380. doi: 10.3389/fpls.2017.01380 (PMC5559707; doi:10.3389/fpls.2017.01380)

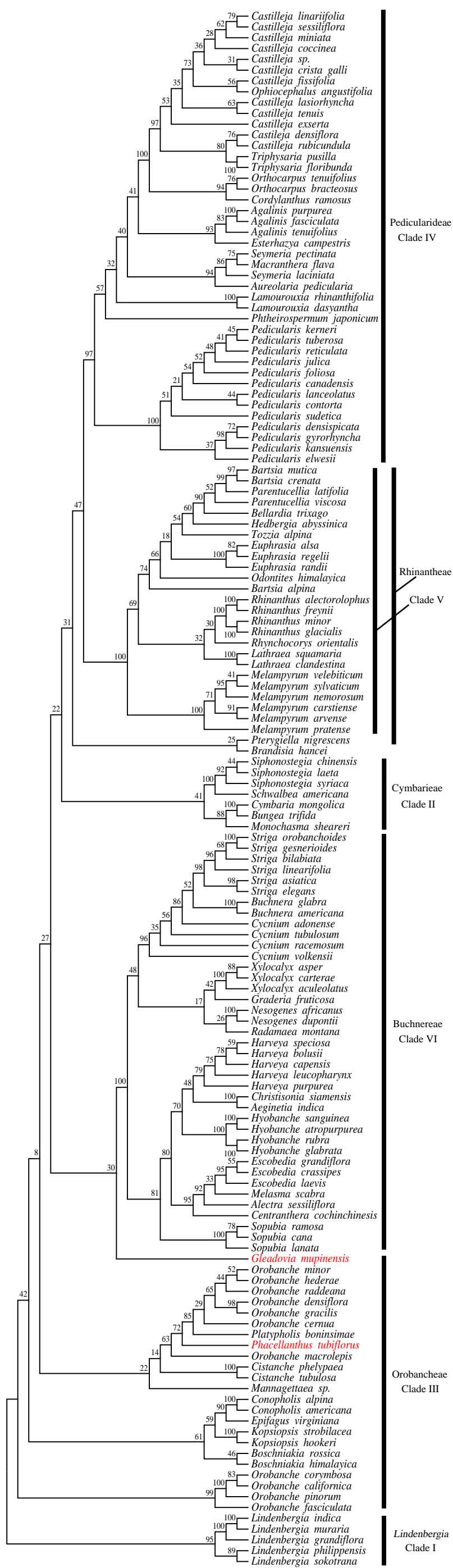

Supplement: Figure S1 — Phylogenetic tree of Orobanchaceae including Gleadovia mupinensis and Phacellanthus tubiflorus based on ITS data with ML method. [file Image1.PDF]

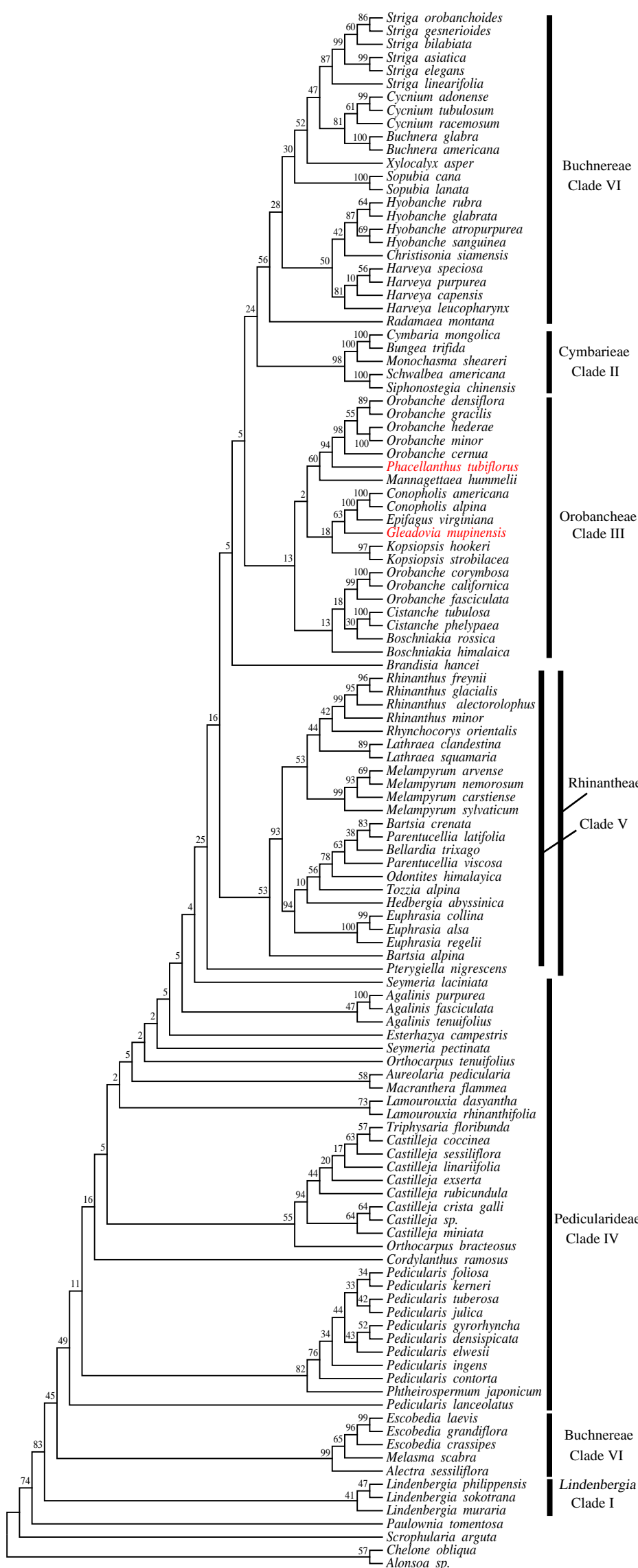

Supplement: Figure S2 — Phylogenetic tree of Orobanchaceae including Gleadovia mupinensis and Phacellanthus tubiflorus based on rps2 data with ML method. [file Image2.PDF]

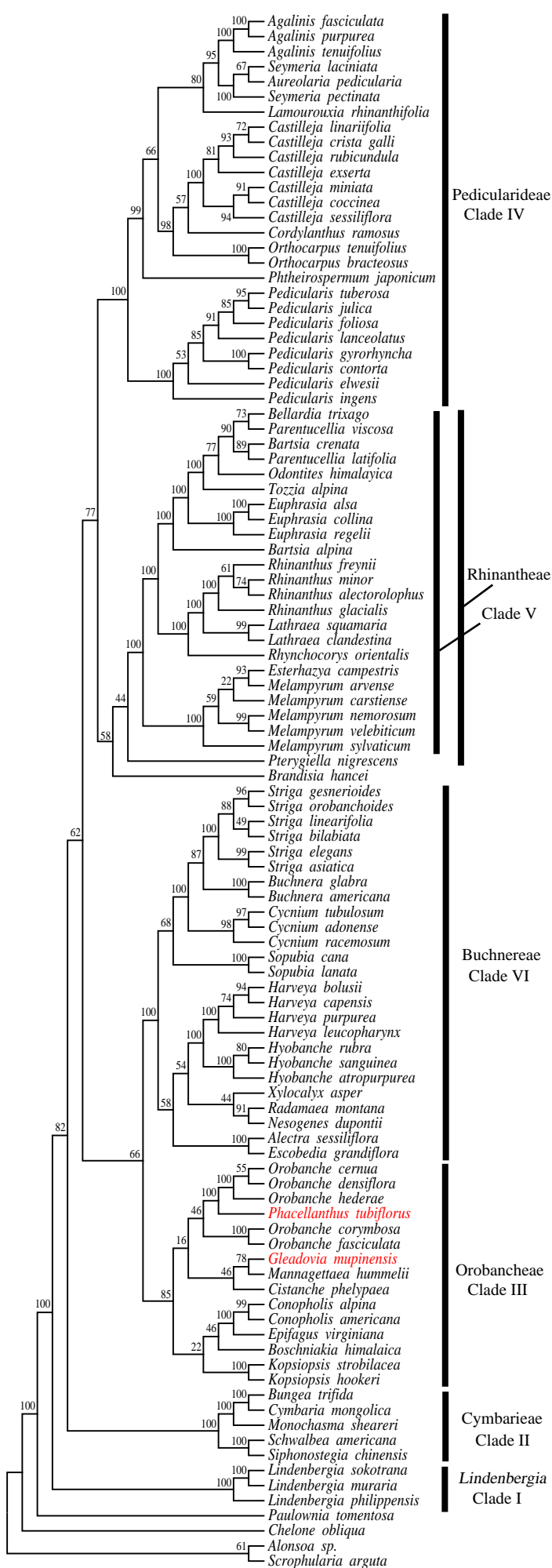

Supplement: Figure S3 — Phylogenetic tree of Orobanchaceae including Gleadovia mupinensis and Phacellanthus tubiflorus based on matK data with ML method. [file Image3.PDF]

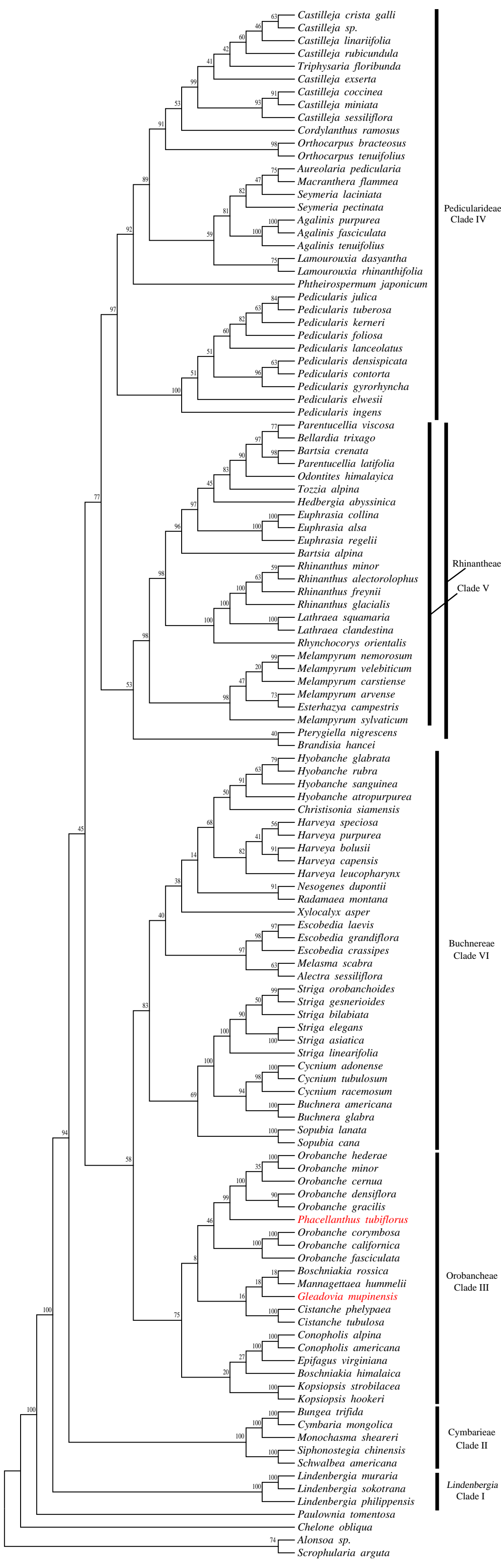

Supplement: Figure S4 — Phylogenetic tree of Orobanchaceae including Gleadovia mupinensis and Phacellanthus tubiflorus based on rps2 + matK data with ML method. [file Image4.PDF]

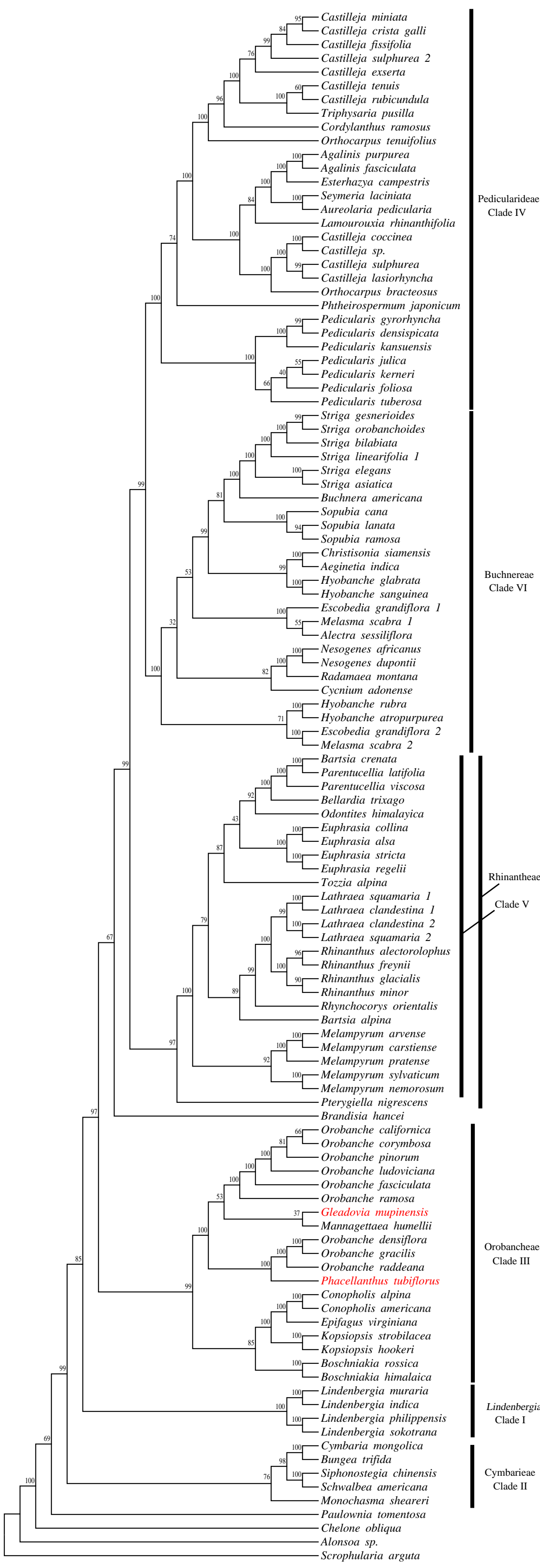

Supplement: Figure S5 — Phylogenetic tree of Orobanchaceae including Gleadovia mupinensis and Phacellanthus tubiflorus based on PHYA data with ML method. [file Image5.PDF]

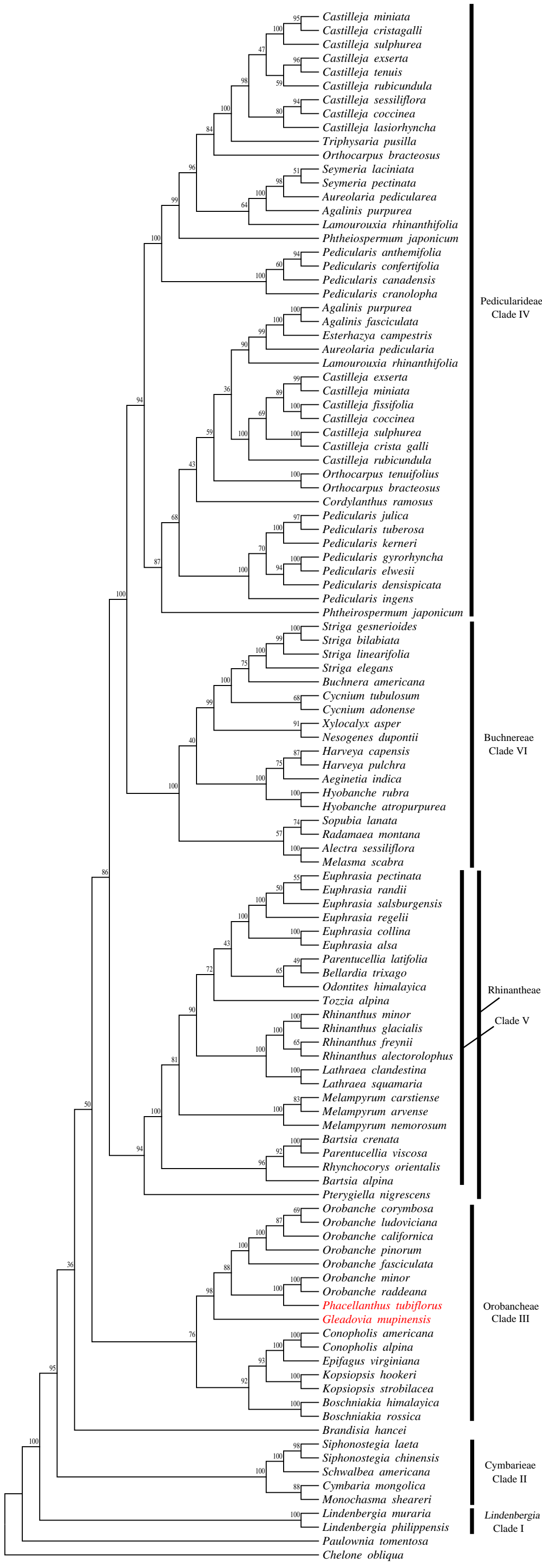

Supplement: Figure S6 — Phylogenetic tree of Orobanchaceae including Gleadovia mupinensis and Phacellanthus tubiflorus based on PHYB data with ML method. [file Image6.PDF]

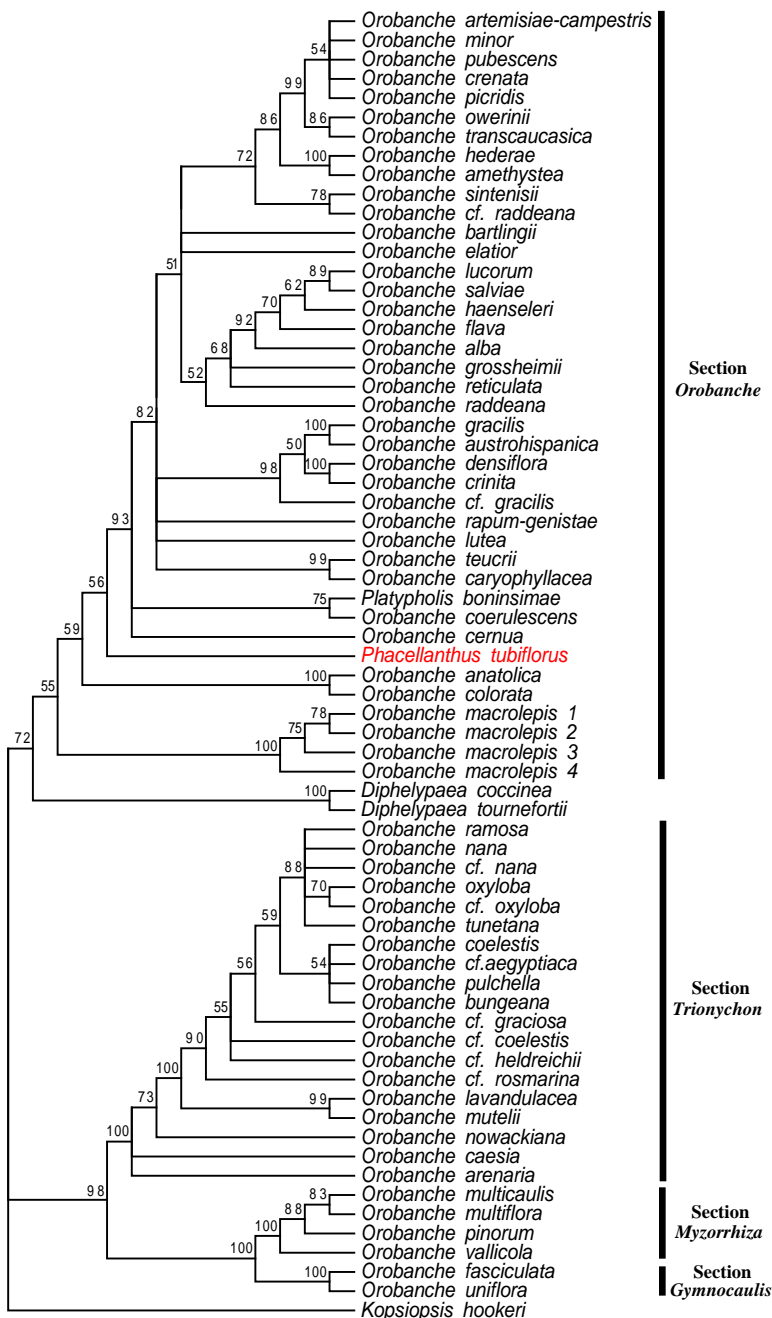

Supplement: Figure S7 — Expanded phylogenetic trees based on ITS sequences for Orobanche with ML method. [file Image7.PDF]

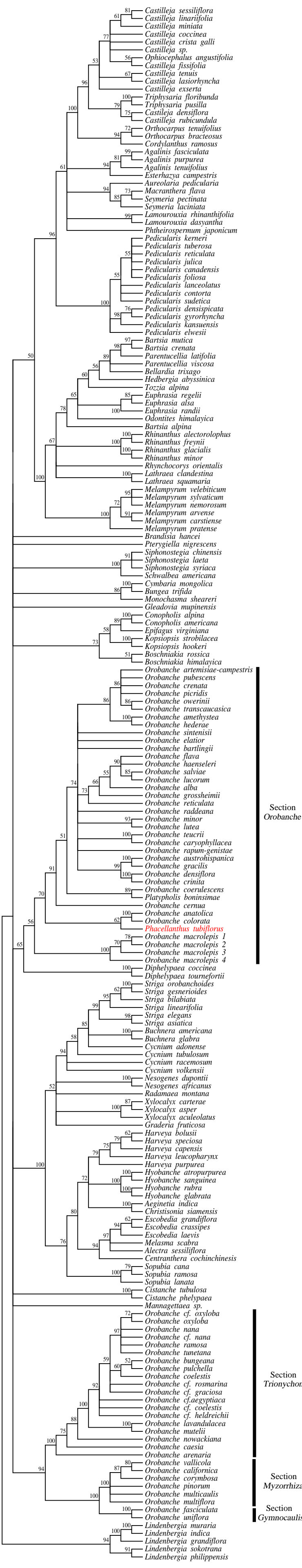

Supplement: Figure S8 — Expanded phylogenetic trees based on ITS sequences for Orobanchaceae with ML method. [file Image8.PDF]

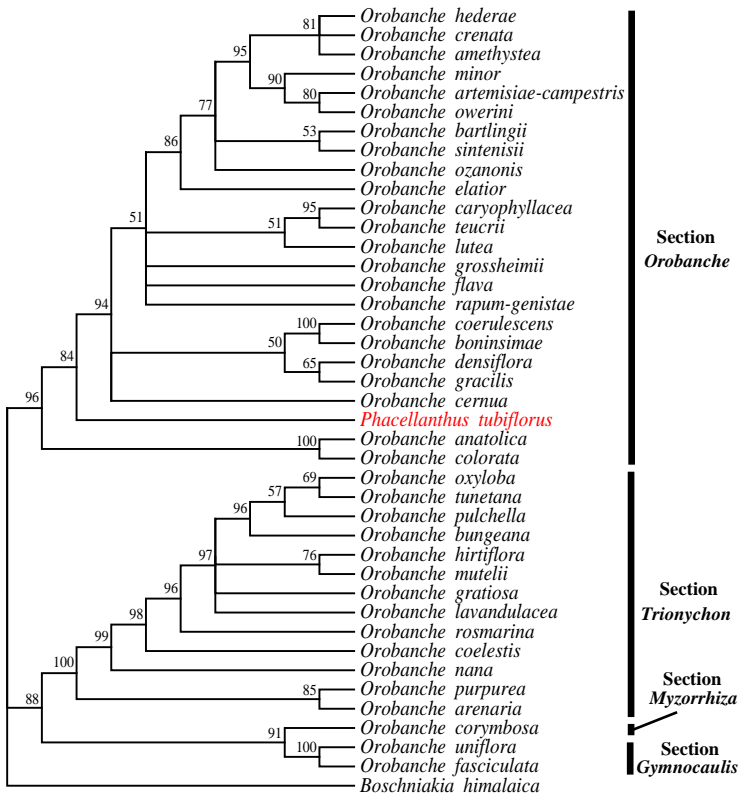

Supplement: Figure S9 — Expanded phylogenetic trees based on rps2 sequences for Orobanche with ML method. [file Image9.PDF]

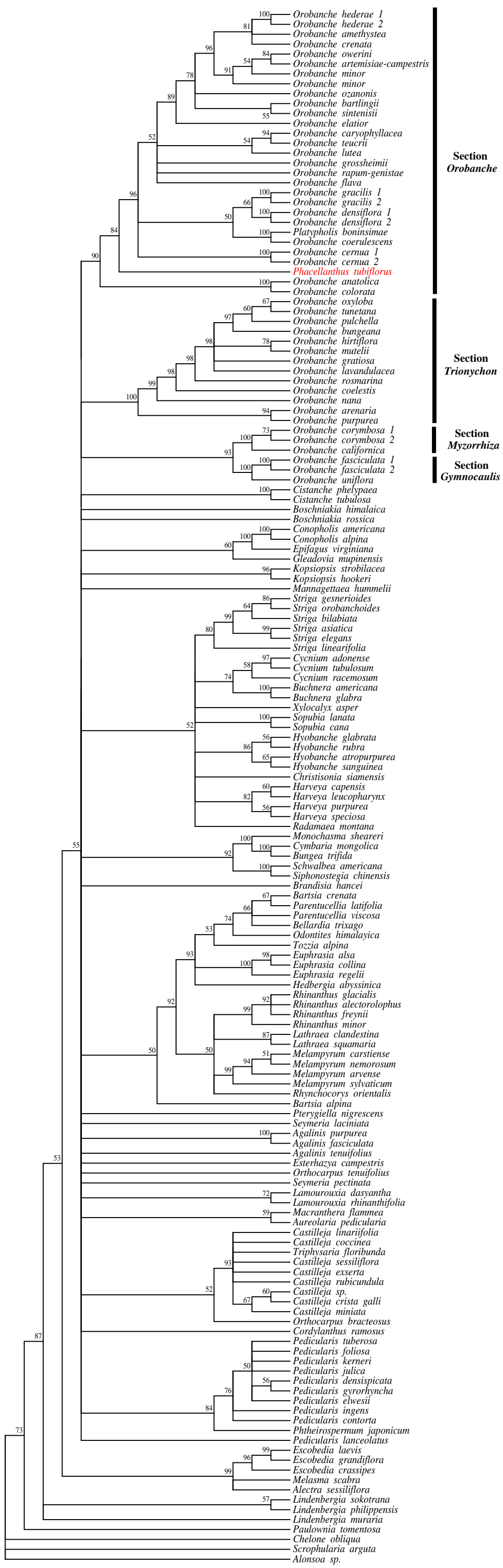

Supplement: Figure S10 — Expanded phylogenetic trees based on rps2 sequences for Orobanchaceae with ML method. [file Image10.PDF]

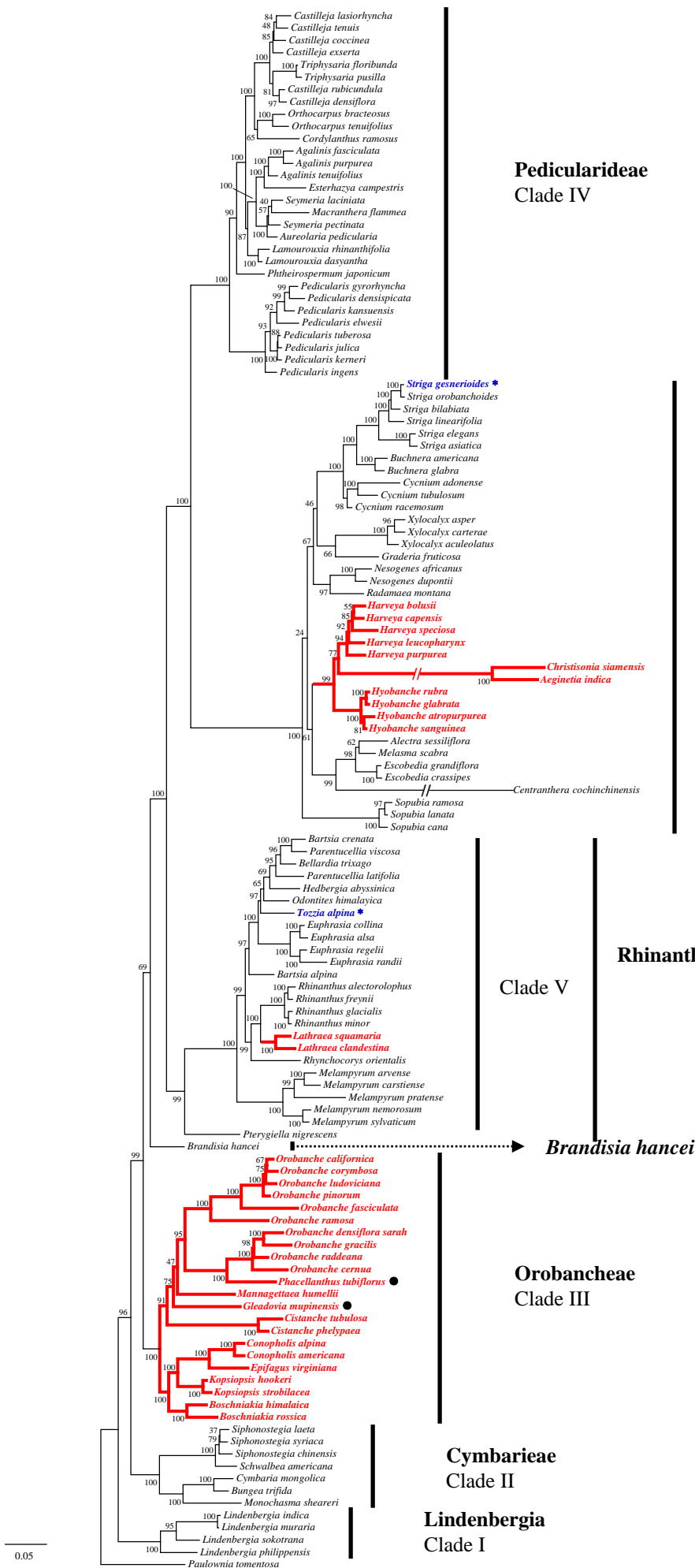

Supplement: Figure S11 — Expanded maximum likelihood phylogenetic tree of Orobanchaceae inferred from the combined five-gene data set (PHYA, PHYB, ITS, matK, and rps2) partitioned by gene. [file Image11.PDF]

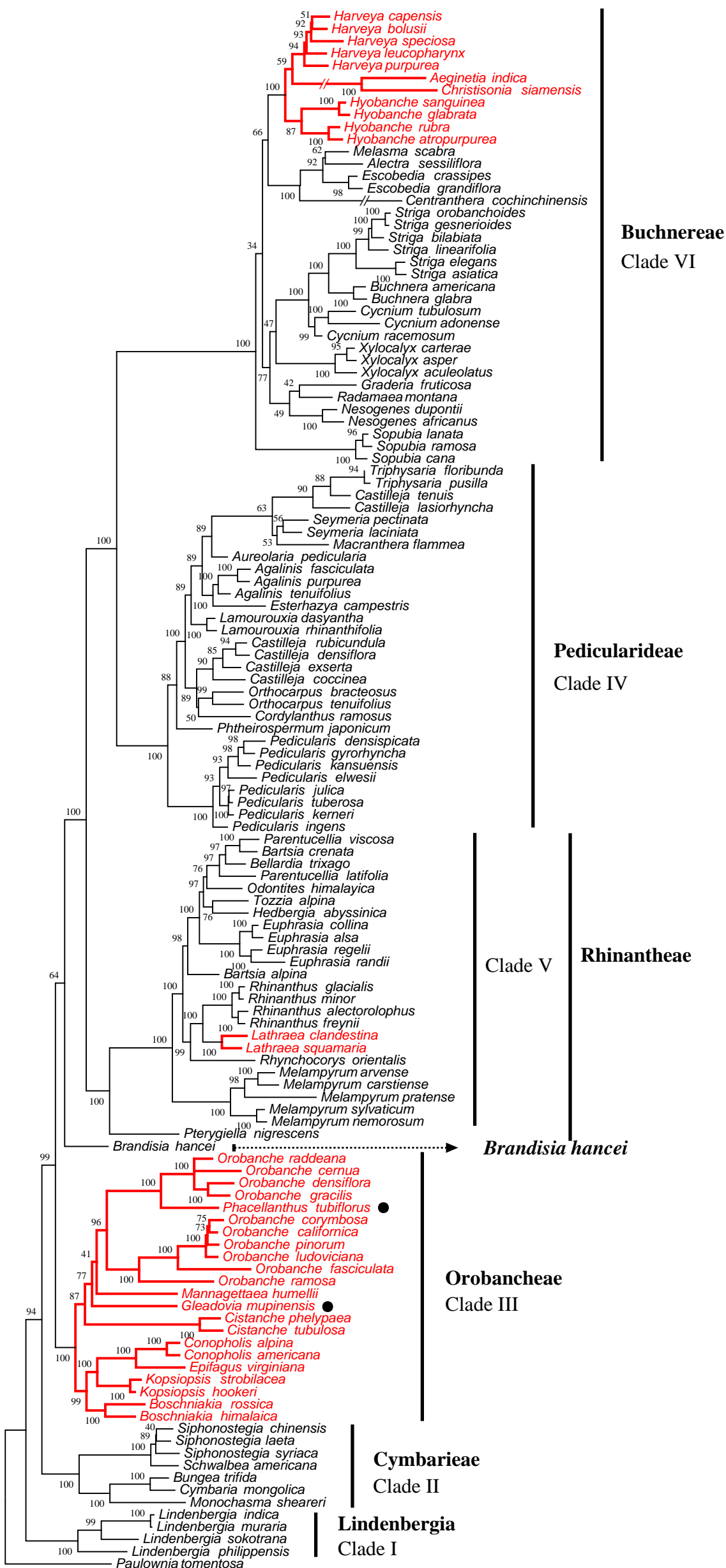

Supplement: Figure S12 — Maximum likelihood phylogenetic tree of Orobanchaceae inferred from the combined five-gene data set (PHYA, PHYB, ITS, matK, and rps2) partitioned by codon. [file Image12.PDF]

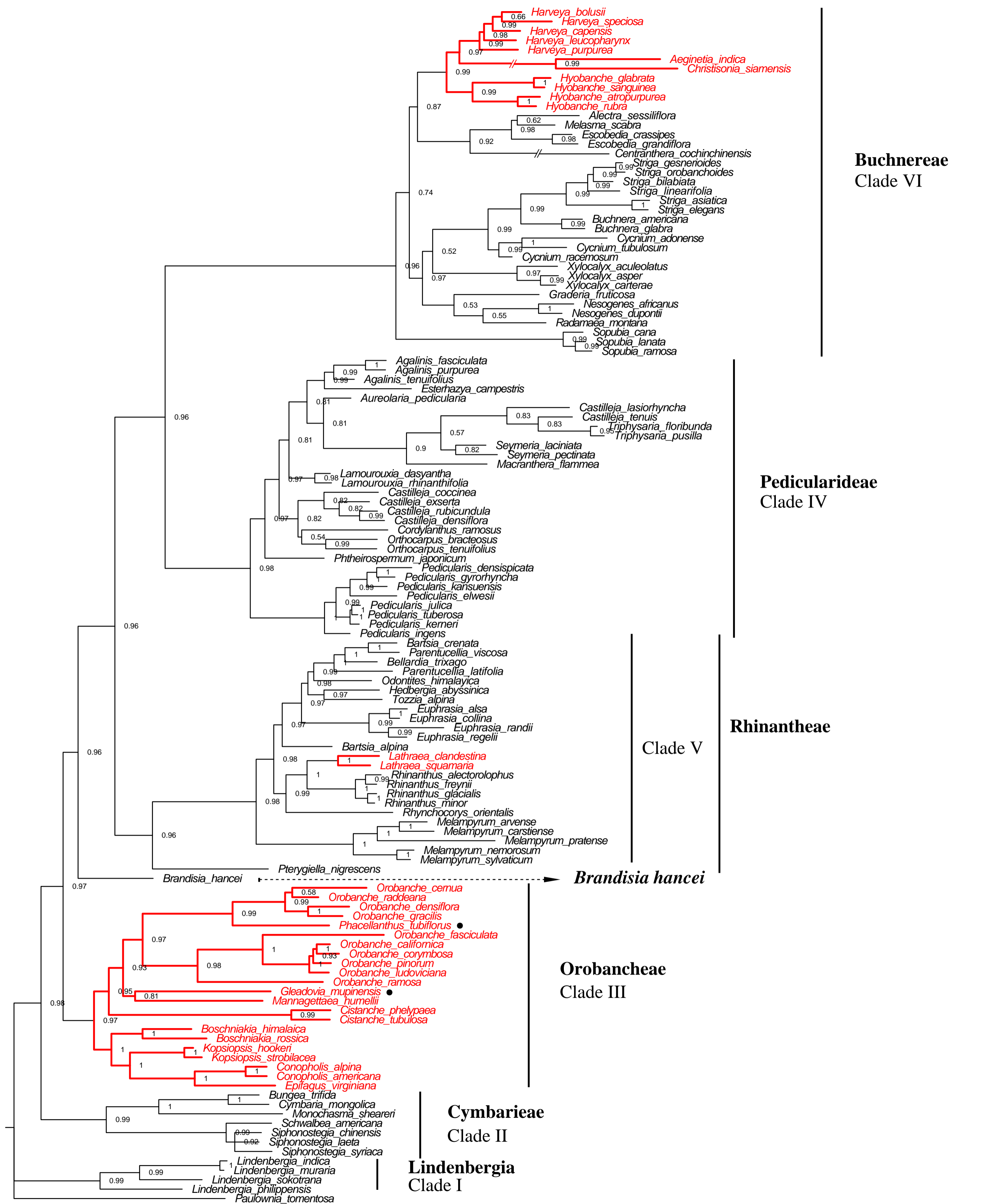

Supplement: Figure S14 — Bayesian phylogenetic tree of Orobanchaceae inferred from the combined five-gene data set (PHYA, PHYB, ITS, matK, and rps2) partitioned by codon. [file Image14.PDF]

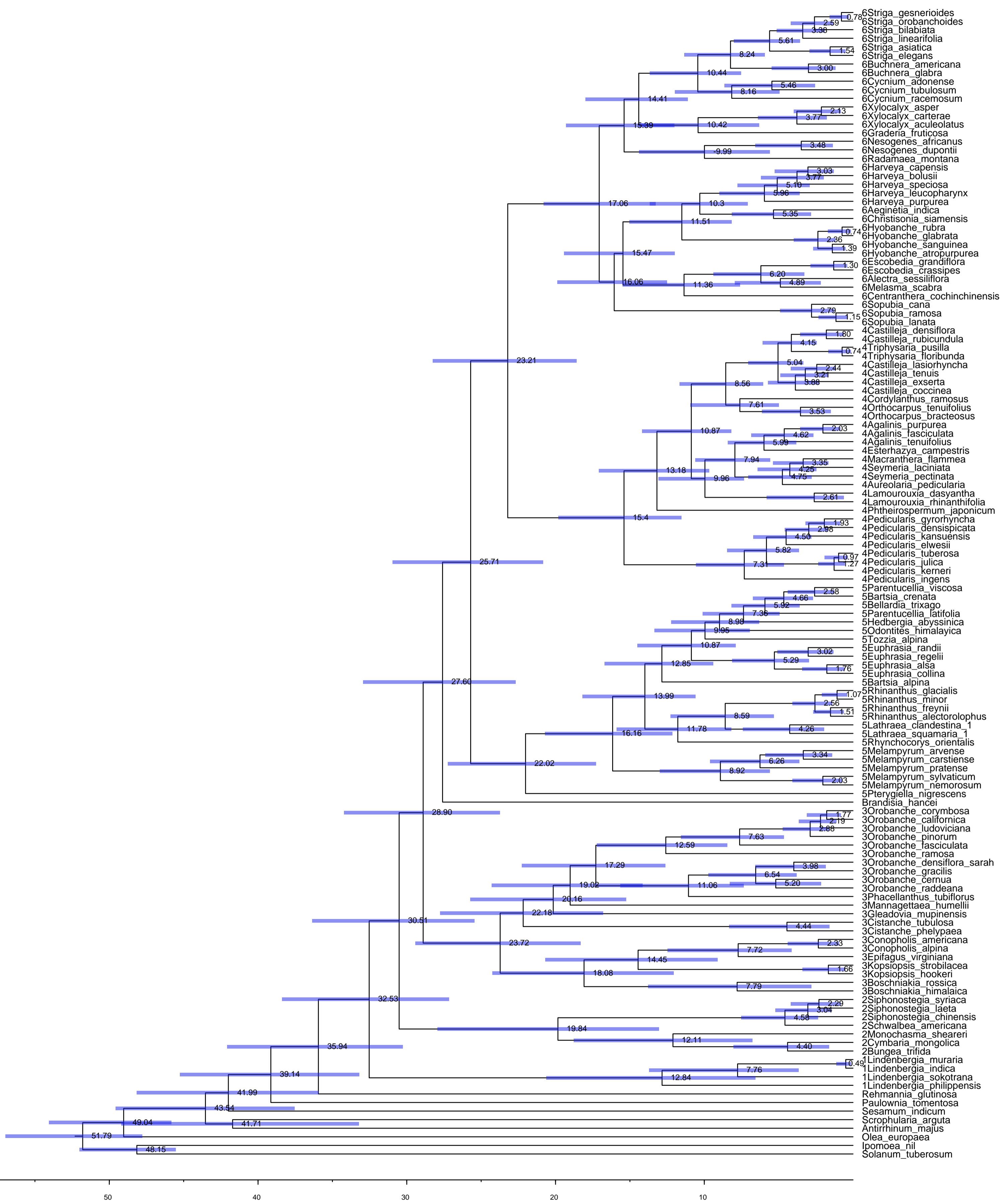

Supplement: Figure S15 — Divergence time estimation of Orobanchaceae using combined five-gene data obtained from BEAST analysis. [file Image15.PDF]
