# Supplementary material for: Testing the Hypothesis of Multiple Origins of Holoparasitism in Orobanchaceae: Phylogenetic Evidence from the Last Two Unplaced Holoparasitic Genera, Gleadovia and Phacellanthus
Source: Front Plant Sci. 2017 Aug 15;8:1380. doi: 10.3389/fpls.2017.01380 (PMC5559707; doi:10.3389/fpls.2017.01380)
Supplement: Figure S13 — Bayesian phylogenetic tree of Orobanchaceae inferred from the combined five-gene data set (PHYA, PHYB, ITS, matK, and rps2) partitioned by gene. [file Image13.PDF]

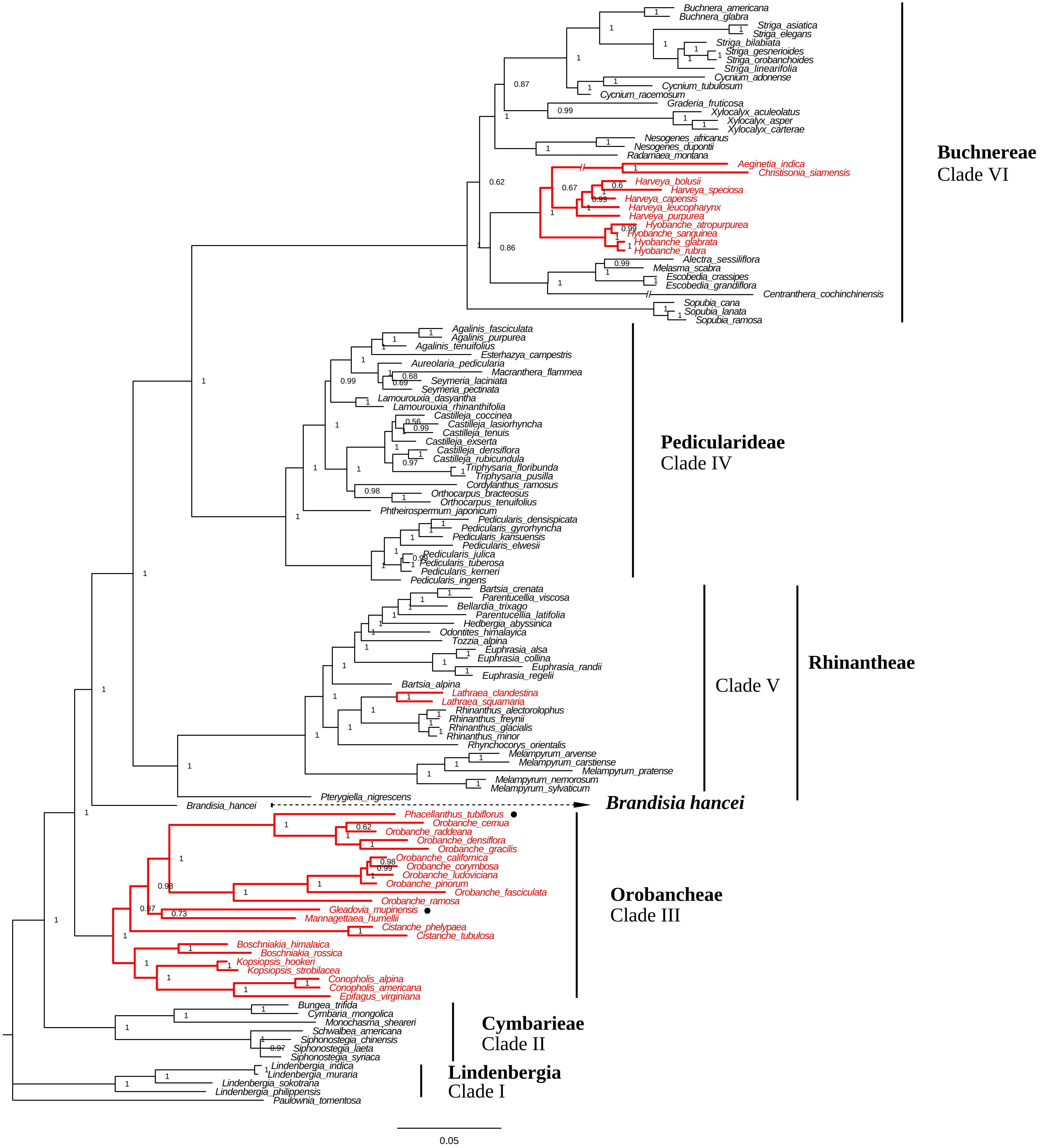
Buchnera_americana
Buchnera_glabra
Striga_asiatica
Striga_elegans
Striga_bilabiata
Striga_gesnerioides
Striga_orobanchoides
Striga_linearifolia
Cycnium_adonense
Cycnium_tubulosum
Cycnium_racemosum
Graderia_fruticosa
Xylocalyx_aculeolatus
Xylocalyx_asper
Xylocalyx_carteræ
Nesogenes_africanus
Nesogenes_dupontii
Radamaea_montana
Aeginetia_indica
Christisonia_siamensis
Harveya_bolusii
Harveya_speciosa
Harveya_capensis
Harveya_leucopharynx
Harveya_purpurea
Hyobanche_atropurpurea
Hyobanche_sanguinea
Hyobanche_glabrata
Hyobanche_rubra
Alectra_sessiliflora
Melasma_scabra
Escobedia_crassipes
Escobedia_grandiflora
Centranthera_cochinchinensis
Sopubia_cana
Sopubia_lanata
Sopubia_ramosa
Buchnereae
Clade VI
Agalinis_fasciculata
Agalinis_purpurea
Agalinis_tenuifolius
Esterhazya_campestris
Aureolaria_pedicularia
Macranthera_flammea
Seymeria_laciniata
Seymeria_pectinata
Lamourouxia_dasyantha
Lamourouxia_rhinanthifolia
Castilleja_coccinea
Castilleja_lasiorhyncha
Castilleja_tenuis
Castilleja_exserta
Castilleja_densiflora
Castilleja_rubicundula
Triphysaria_floribunda
Triphysaria_pusilla
Cordylanthus_ramosus
Orthocarpus_bracteosus
Orthocarpus_tenuifolius
Phtheirospermum_japonicum
Pedicularis_densispicata
Pedicularis_gyrorhyncha
Pedicularis_kansuensis
Pedicularis_elwesii
Pedicularis_julica
Pedicularis_tuberosa
Pedicularis_kerneri
Pedicularis_ingens
Pedicularideae
Clade IV
Bartsia_crenata
Parentucellia_viscosa
Bellardia_trixago
Parentucellia_latifolia
Hedbergia_abyssinica
Odontites_himalayica
Tozzia_alpina
Euphrasia_alsa
Euphrasia_collina
Euphrasia_randii
Euphrasia_regelii
Bartsia_alpina
Lathraea_clandestina
Lathraea_squamaria
Rhinanthus_alectorolophus
Rhinanthus_freynii
Rhinanthus_glacialis
Rhinanthus_minor
Rhynchocorys_orientalis
Melampyrum_arvense
Melampyrum_carstiense
Melampyrum_pratense
Melampyrum_nemorosum
Melampyrum_sylvaticum
Pterygiella_nigrescens
Clade V
Rhinantheae
Brandisia_hancei
Brandisia hancei
Phacellanthus_tubiflorus
Orobanche_cernua
Orobanche_raddeana
Orobanche_densiflora
Orobanche_gracilis
Orobanche_californica
Orobanche_corymbosa
Orobanche_ludoviciana
Orobanche_pinorum
Orobanche_fasciculata
Orobanche_ramosa
Gleadovia_mupinensis
Mannagettaea_hummellii
Cistanche_phelypaea
Cistanche_tubulosa
Boschniakia_himalaica
Boschniakia_rossica
Kopsiopsis_hookeri
Kopsiopsis_strobilacea
Conopholis_alpina
Conopholis_americana
Epifagus_virginiana
Orobancheae
Clade III
Bungea_trifida
Cymbaria_mongolica
Monochasma_sheareri
Schwalbea_americana
Siphonostegia_chinensis
Siphonostegia_laeta
Siphonostegia_syriaca
Cymbarieae
Clade II
Lindenbergia_indica
Lindenbergia_muraria
Lindenbergia_sokotrana
Lindenbergia_philippensis
Paulownia_tomentosa
Lindenbergia
Clade I
0.05
