# Supplementary material for: Testing the Hypothesis of Multiple Origins of Holoparasitism in Orobanchaceae: Phylogenetic Evidence from the Last Two Unplaced Holoparasitic Genera, Gleadovia and Phacellanthus
Source: Front Plant Sci. 2017 Aug 15;8:1380. doi: 10.3389/fpls.2017.01380 (PMC5559707; doi:10.3389/fpls.2017.01380)
Supplement: Table S1 — The primer information for PCR amplification and cycle sequencing. [file Table1.DOCX]

**Table S1** The primer information for PCR amplification and cycle sequencing.

| **Gene ID Primer name** | | **Primer sequence** | **Reference** |
| --- | --- | --- | --- |
| ITS | ITS-5F | 5'-GGAAGTAAAAGTCGTAACAAGG-3' | White *et al.*, 1990 |
|  | ITS-4R | 5'-TCCTCCGCTTATTGATATGC-3' | White *et al.*, 1990 |
| *rps2* | *rps2*-18F | 5'-GGRKARAAATGACAAGAAGATATTGG-3' | dePamphilis *et al.*, 1997 |
|  | *rps2*-661R | 5'-ACCCTCACAAATAGCGAATACCAA-3' | dePamphilis *et al.*, 1997 |
| *mat*K | *mat*K-1F | 5'-ACTGTATCGACATATGTATCA-3' | Sang *et al.*, 1997 |
|  | *mat*K-1R | 5'-GAACTAGTCCGATGGAGTAG-3' | Sang *et al.*, 1997 |
| *PHYA* | *PHYA*-1F | 5'-GCTTTGCGATACTATGGTGC-3' | This study |
|  | *PHYA*-1R | 5'-CGATTCTCGTGAACTTGTCC-3' | This study |
|  | *PHYA*-2R | 5'-TGCGATGAAACATACTCCTAC-3' | This study |
| *PHYB* | *PHYB*-1F | 5'-ATGGTGAGGTTGTGGCAGAG-3' | This study |
|  | *PHYB*-1R | 5'-CCGATTAGCATTTTCCCG-3' | This study |
|  | *PHYB*-2F | 5'-AGCCTTTGTGTTTGGTTGG-3' | This study |
|  | *PHYB*-2R | 5'-TCTTGCTGGTTTCAGTGCC-3' | This study |

References

Sang, T., Crawford, D. J., Stuessy, T. F. (1997). Chloroplast DNA phylogeny, reticulate evolution, and biogeography of *Paeonia* (Paeoniaceae). *Amer. J. Bot.* 84, 1120–1136.

White, T. J., Bruns, T., Lee, S., Taylor, W. J. (1990). “Amplification and direct sequencing of fungal ribosomal RNA genes for phylogenetics.” in: Innis, M. A., Gelfand, D. H., Sninsky, J. J., White, T. J. (Eds.), *PCR Protocols: A Guide to Methods and Applications*. (Academic Press, San Diego), 315–322.

dePamphilis, C. W., Young, N. D., Wolfe, A. D. (1997). Evolution of plastid gene *rps2* in a lineage of hemiparasitic and holoparasitic plants: many losses of photosynthesis and complex patterns of rate variation. *Proc. Nati. Acad. Sci. USA.* 94, 7367–7372.
