# Supplementary material for: Testing the Hypothesis of Multiple Origins of Holoparasitism in Orobanchaceae: Phylogenetic Evidence from the Last Two Unplaced Holoparasitic Genera, Gleadovia and Phacellanthus
Source: Front Plant Sci. 2017 Aug 15;8:1380. doi: 10.3389/fpls.2017.01380 (PMC5559707; doi:10.3389/fpls.2017.01380)
Supplement: Table S2 — PCR amplification programs of each gene used in this study. [file Table2.DOCX]

**Table S2** PCR amplification programs of each gene used in this study.

| Gene | Denaturation time | Annealing time | Extension time | Annealing temperature |
| --- | --- | --- | --- | --- |
| ITS | 30s | 45s | 60s | 54°C |
| *rps2* | 30s | 45s | 60s | 53°C |
| *mat*K | 30s | 120s | 120s | 51°C |
| *PHYA* | 60s | 120s | 150s | 53°C |
| *PHYB* | 60s | 120s | 150s | 53°C |
