# Supplementary material for: Testing the Hypothesis of Multiple Origins of Holoparasitism in Orobanchaceae: Phylogenetic Evidence from the Last Two Unplaced Holoparasitic Genera, Gleadovia and Phacellanthus
Source: Front Plant Sci. 2017 Aug 15;8:1380. doi: 10.3389/fpls.2017.01380 (PMC5559707; doi:10.3389/fpls.2017.01380)
Supplement: Table S3 — GenBank accession numbers for species used in this study. [file Table3.DOCX]

**Table S3** GenBank accession numbers for species used in this study.

| **Species** | **ITS** | ***rps2*** | ***mat*K** | ***PHYA*** | ***PHYB*** |
| --- | --- | --- | --- | --- | --- |
| *Aeginetia indica* | KC480292 | N/A | N/A | AM233920 | KC542196 |
| *Agalinis fasciculata* | KC480293 | JX02297 | AY563919 | AM233921 | KC542197 |
| *Agalinis purpurea* | KC480294 | JX022971 | JX091307 | AM233922 | KC542198 |
| *Agalinis tenuifolia* | KC480295 | KC480413 | AY563916 | N/A | N/A |
| *Alectra sessiliflora* | KC480296 | JX022972 | JX091308 | AM233923 | KC542199 |
| *Alonsoa sp.* | N/A | JX022973 | JX091309 | AM237453 | N/A |
| *Antirrhinum majus* | N/A | AMU48766 | AF051978 | N/A | N/A |
| *Aureolaria pedicularia* | KC480297 | EU828043 | AY563920 | AM233926 | KC542200 |
| *Bartsia alpina* | KC480298 | U48751 | AY849600 | AM233928 | KC542201 |
| *Bartsia crenata* | KC480299 | KC480414 | KC542148 | AM233927 | KC542202 |
| *Bartsia mutica* | KC480300 | N/A | N/A | N/A | N/A |
| *Bellardia trixago* | KC480301 | JX022974 | JX091310 | AM233930 | KC542203 |
| *Boschniakia himalaica* | AY911212 | JX022975 | JX091311 | KC542187 | KC542204 |
| *Boschniakia rossica* | KC480303 | KC480415 | N/A | AM233932 | KC542206 |
| *Brandisia hancei* | KC480305 | JX022978 | KC542149 | AM233934 | KC542208 |
| *Buchnera americana* | KC480306 | U48744 | KC542150 | KC542188 | KC542209 |
| *Buchnera glabrata* | AY911216 | JX022979 | KC556899 | N/A | N/A |
| *Bungea trifida* | KC480307 | JX022980 | KC542151 | AM233936 | N/A |
| *Castilleja coccinea* | KC480308 | JX022981 | JX091314 | AM233938 | KC542210 |
| *Castilleja crista-galli* | KC480309 | KC480416 | JX091315 | AM233937 | KC542211 |
| *Castilleja densiflora* | KC480310 | N/A | N/A | N/A | N/A |
| *Castilleja exserta* | KC480311 | JX022982 | KC542152 | AM233939 | KC542212 |
| *Castilleja fissifolia* | KC480312 | N/A | N/A | AM233944 | KC542213 |
| *Castilleja lasiorhyncha* | KC480313 | N/A | N/A | AM233940 | N/A |
| *Castilleja linariifolia* | KC480314 | U48739 | AF051981 | N/A | N/A |
| *Castilleja miniata* | KC480315 | JX022983 | KC542153 | AM233941 | KC542214 |
| *Castilleja rubicundula* | KC480316 | JX022984 | KC542154 | AM233942 | KC542215 |
| *Castilleja sessiliflora* | KC480317 | JX022985 | JX091316 | N/A | N/A |
| *Castilleja sp.* | KC480318 | JX022986 | N/A | N/A | N/A |
| *Castilleja sulphurea* | N/A | N/A | N/A | AM233945 | KC542281 |
| *Castilleja tenuis* | KC480319 | N/A | N/A | AM233947 | N/A |
| *Centranthera cochinchinensis* | KC480320 | N/A | N/A | N/A | N/A |
| *Chelone obliqua* | N/A | U48770 | KC542147 | AM233948 | KC542194 |
| *Christisonia siamensis* | KC480321 | KC480417 | N/A | KC542189 | N/A |
| *Cistanche phelypaea* | KC480322 | JX022987 | JX091317 | N/A | N/A |
| *Cistanche tubulosa* | KC480323 | AB062409 | N/A | N/A | N/A |
| *Conopholis alpina* | KC480324 | JX022988 | KC542155 | AM233949 | KC542216 |
| *Conopholis americana* | KC480325 | JX022989 | KC542156 | AM233950 | KC542217 |
| *Cordylanthus ramosus* | KC480326 | JX022990 | KC542157 | AM233951 | KC556900 |
| *Cycnium adonense* | KC480327 | JX022991 | JX091318 | AM233952 | KC542218 |
| *Cycnium racemosum* | KC480328 | JX022992 | JX091319 | N/A | N/A |
| *Cycnium tubulosum* | KC480329 | KC480418 | JX091320 | N/A | KC542219 |
| *Cycnium volkensii* | KC480330 | N/A | N/A | N/A | N/A |
| *Cymbaria mongolica* | KC480331 | KC480419 | KC542158 | AM233953, AM234038 | KC542220 |
| *Diphelypaea tournefortii* | AY209285 | N/A | N/A | N/A | N/A |
| *Diphelypaea coccinea* | AY209284 | N/A | N/A | N/A | N/A |
| *Epifagus virginiana* | KC480332 | JX022993 | AF051982 | AM234039 | KC542221 |
| *Escobedia crassipes* | KC480333 | JX022994 | N/A | N/A | N/A |
| *Escobedia grandiflora* | KC480334 | JX022995 | JX091321 | AM233955 | N/A |
| *Escobedia laevis* | KC480335 | KC480420 | N/A | N/A | N/A |
| *Esterhazya campestris* | KC480336 | JX022996 | KC542159 | AM233957 | KC542222 |
| *Euphrasia alsa* | KC480337 | KC480421 | KC542160 | AM233958 | KC542223 |
| *Euphrasia collina* | N/A | JX022997 | KC542161 | AM233959 | KC542224 |
| *Euphrasia pectinata* | N/A | N/A | N/A | N/A | KC542225 |
| *Euphrasia randii* | KC480338 | N/A | N/A | N/A | KC542226 |
| *Euphrasia regelii* | KC480339 | JX022998 | KC542162 | AM233960 | KC542227 |
| *Euphrasia salisburgensis* | N/A | JX022999 | JX091322 | N/A | KC542228 |
| *Euphrasia spectabilis* | N/A | N/A | AY849603 | N/A | N/A |
| *Euphrasia stricta* | N/A | N/A | N/A | AM233961 | N/A |
| *Gleadovia mupinensis* | KY706614 | KY706619 | KY706624 | KY706629 | KY706631 |
| *Graderia fruticosa* | KC480340 | N/A | N/A | N/A | N/A |
| *Harveya bolusii* | KC480341 | N/A | AF051983 | N/A | N/A |
| *Harveya capensis* | KC480342 | JX023000 | AF489961 | N/A | KC542229 |
| *Harveya leucopharynx* | KC480343 | JX023001 | JX091323 | N/A | N/A |
| *Harveya purpurea* | KC480344 | JX023002 | AF051984 | N/A | N/A |
| *Harveya speciosa* | AY911228 | KC480422 | N/A | N/A | N/A |
| *Hedbergia abyssinica* | KC480345 | JX023003 | N/A | N/A | N/A |
| *Hyobanche atropurpurea* | KC480346 | JX023004 | AF051986 | AM233964 | KC542230 |
| *Hyobanche glabrata* | KC480347 | JX023005 | N/A | AM233965 | N/A |
| *Harveya pulchra* | N/A | N/A | N/A | N/A | KC542280 |
| *Hyobanche rubra* | KC480348 | JX023006 | JX091324 | AM233966 | KC542231 |
| *Hyobanche sanguinea* | KC480349 | JX023007 | AF051987 | AM233967 | N/A |
| *Ipomoea nil* | KR052191 | AP017304 | AP017304 | N/A | XM_019319246 |
| *Kopsiopsis hookeri* | KC480302 | JX022976 | JX091312 | AM233931 | KC542205 |
| *Kopsiopsis strobilacea* | KC480304 | JX022977 | JX091313 | AM233933 | KC542207 |
| *Lamourouxia dasyantha* | KC480350 | KC480423 | N/A | N/A | N/A |
| *Lamourouxia rhinanthifolia* | KC480351 | JX023008 | KC542163 | AM233969 | KC542232 |
| *Lathraea clandestina* | KC480352 | JX023009 | JX091325 | AM233970 | KC542233 |
| *Lathraea squamaria* | KC480353 | KC480424 | KC542164 | AM233972 | KC542234 |
| *Lindenbergia grandiflora* | KC480287 | N/A | N/A | N/A | N/A |
| *Lindenbergia indica* | KC480288 | N/A | N/A | AM233976 | N/A |
| *Lindenbergia muraria* | KC480289 | JX023010 | KC542165 | AM233977 | KC542235 |
| *Lindenbergia philippensis* | KC480290 | JX023011 | AF051990 | AM233978 | KC542236 |
| *Lindenbergia sokotrana* | KC480291 | KC480425 | JX091326 | AM233918 | N/A |
| *Macranthera flammea* | KC480354 | AF055139 | N/A | N/A | N/A |
| *Mannagettaea hummelii* | KC480355 | KC480426 | KC542166 | KC542190 | N/A |
| *Melampyrum arvense* | KC480356 | JX023012 | JX091327 | AM233919 | KC542237 |
| *Melampyrum carstiense* | KC480357 | JX023013 | KC542167 | AM233979 | KC542238 |
| *Melampyrum nemorosum* | KC480358 | JX023014 | KC542168 | AM233985 | KC542239 |
| *Melampyrum pratense* | KC480359 | N/A | N/A | AM233981 | N/A |
| *Melampyrum sylvaticum* | KC480360 | JX023015 | AF051991 | AM233982 | N/A |
| *Melampyrum velebiticum* | KC480361 | N/A | N/A | N/A | N/A |
| *Melasma scabrum* | KC480362 | JX023016 | AY849604 | AM233984 | KC542240 |
| *Monochasma sheareri* | KC480363 | KC480427 | KC542169 | AM233985, AM234042 | KC542241 |
| *Nesogenes africanus* | KC480364 | N/A | N/A | AM233986 | N/A |
| *Nesogenes dupontii* | KC480365 | N/A | KC542170 | AM233987 | KC542242 |
| *Odontites himalayica* | KC480366 | KC480428 | KC542171 | AM233988 | KC542243 |
| *Olea europaea* | KF805102 | GU931818 | GU931818 | AY924378 | N/A |
| *Ophiocephalus angustifolius* | KC480367 | N/A | N/A | N/A | N/A |
| *Orobanche alba* | AY209245 | N/A | N/A | N/A | N/A |
| *Orobanche amethystea* | AY209274 | DQ310023 | N/A | N/A | N/A |
| *Orobanche anatolica* | AY209282 | DQ310013 | N/A | N/A | N/A |
| *Orobanche arenaria* | AY209346 | DQ310047 | N/A | N/A | N/A |
| *Orobanche artemisiae-campestris* | AY209264 | DQ310018 | N/A | N/A | N/A |
| *Orobanche austrohispanica* | AY209241 | N/A | N/A | N/A | N/A |
| *Orobanche bartlingii* | AY209260 | DQ310017 | N/A | N/A | N/A |
| *Orobanche bungeana* | AY209330 | DQ310110 | N/A | N/A | N/A |
| *Orobanche caesia* | AY209351 | N/A | N/A | N/A | N/A |
| *Orobanche californica* | KC480368 | KC480429 | N/A | AM233989 | KC542244 |
| *Orobanche caryophyllacea* | AY209237 | AF055145 | N/A | N/A | N/A |
| *Orobanche cernua* | AY911235 | AF055147 | AF056147 | N/A | N/A |
| *Orobanche* cf. *aegyptiaca* | AY209327 | N/A | N/A | N/A | N/A |
| *Orobanche* cf. *coelestis* | AY209332 | N/A | N/A | N/A | N/A |
| *Orobanche* cf. *gracilis* | AY209242 | N/A | N/A | N/A | N/A |
| *Orobanche* cf. *graciosa* | AY209331 | N/A | N/A | N/A | N/A |
| *Orobanche* cf. *heldreichii* | AY209333 | N/A | N/A | N/A | N/A |
| *Orobanche* cf. *nana* | AY209314 | N/A | N/A | N/A | N/A |
| *Orobanche* cf. *oxyloba* | AY209322 | N/A | N/A | N/A | N/A |
| *Orobanche* cf. *raddeana* | AY209259 | N/A | N/A | N/A | N/A |
| *Orobanche* cf. *rosmarina* | AY209334 | N/A | N/A | N/A | N/A |
| *Orobanche coelestis* | AY209329 | DQ310050 | N/A | N/A | N/A |
| *Orobanche coerulescens* | AY209235 | DQ310016 | N/A | N/A | N/A |
| *Orobanche colorata* | AY209281 | DQ310012 | N/A | N/A | N/A |
| *Orobanche corymbosa* | KC480369 | U48760 | AF051993 | AM233990 | KC542245 |
| *Orobanche crenata* | AY209266 | DQ310022 | N/A | N/A | N/A |
| *Orobanche crinita* | AY209244 | N/A | N/A | N/A | N/A |
| *Orobanche densiflora* | KC480370 | N/A | N/A | N/A | N/A |
| *Orobanche elatior* | AY209275 | DQ403781 | N/A | N/A | N/A |
| *Orobanche fasciculata* | KC480371 | AF055143 | AF051994 | AM233992 | KC542248 |
| *Orobanche flava* | AY209254 | DQ310029 | N/A | N/A | N/A |
| *Orobanche gracilis* | KC480372 | N/A | N/A | AM233993 | N/A |
| *Orobanche gratiosa* | N/A | DQ310117 | N/A | N/A | N/A |
| *Orobanche grossheimii* | AY209278 | DQ310028 | N/A | N/A | N/A |
| *Orobanche haenseleri* | AY209253 | N/A | N/A | N/A | N/A |
| *Orobanche hederae* | AY911240 | AF055146 | AF051995 | N/A | N/A |
| *Orobanche hirtiflora* | N/A | DQ310115 | N/A | N/A | N/A |
| *Orobanche lavandulacea* | AY209342 | DQ310122 | N/A | N/A | N/A |
| *Orobanche lucorum* | AY209251 | N/A | N/A | N/A | N/A |
| *Orobanche ludoviciana* | N/A | N/A | N/A | KC542191 | KC542246 |
| *Orobanche lutea* | AY209279 | DQ310025 | N/A | N/A | N/A |
| *Orobanche macrolepi 1* | AY209286 | N/A | N/A | N/A | N/A |
| *Orobanche macrolepis 2* | AY209287 | N/A | N/A | N/A | N/A |
| *Orobanche macrolepis 3* | AY209286 | N/A | N/A | N/A | N/A |
| *Orobanche macrolepis 4* | AY209289 | N/A | N/A | N/A | N/A |
| *Orobanche minor* | KC480373 | N/A | N/A | N/A | KC542249 |
| *Orobanche multicaulis* | AY209294 | N/A | N/A | N/A | N/A |
| *Orobanche multiflora* | AY209293 | N/A | N/A | N/A | N/A |
| *Orobanche mutelii* | AY209337 | DQ310121 | N/A | N/A | N/A |
| *Orobanche nana* | AY209313 | DQ310131 | N/A | N/A | N/A |
| *Orobanche nowackiana* | AY209352 | N/A | N/A | N/A | N/A |
| *Orobanche owerinii* | AY209265 | DQ310020 | N/A | N/A | N/A |
| *Orobanche ozanonis* | N/A | DQ310027 | N/A | N/A | N/A |
| *Orobanche oxyloba* | AY209319 | DQ310108 | N/A | N/A | N/A |
| *Orobanche picridis* | AY209269 | N/A | N/A | N/A | N/A |
| *Orobanche pinorum* | AY209292 | N/A | N/A | AM233994 | KC542247 |
| *Orobanche pubescens* | AY209268 | N/A | N/A | N/A | N/A |
| *Orobanche pulchella* | AY209326 | DQ310055 | N/A | N/A | N/A |
| *Orobanche purpurea* | N/A | DQ310035 | N/A | N/A | N/A |
| *Orobanche raddeana* | AY209258 | N/A | N/A | AM233995 | KC542250 |
| *Orobanche ramosa* | N/A | N/A | N/A | AM23399 | N/A |
| *Orobanche rapum-genistae* | AY209280 | DQ310031 | N/A | N/A | N/A |
| *Orobanche reticulata* | AY209256 | N/A | N/A | N/A | N/A |
| *Orobanche rosmarina* | N/A | DQ310064 | N/A | N/A | N/A |
| *Orobanche salviae* | AY209252 | N/A | N/A | N/A | N/A |
| *Orobanche sintenisii* | AY209276 | DQ310026 | N/A | N/A | N/A |
| *Orobanche teucrii* | AY209236 | DQ310024 | N/A | N/A | N/A |
| *Orobanche transcaucasica* | AY209262 | N/A | N/A | N/A | N/A |
| *Orobanche tunetana* | AY209324 | DQ310069 | N/A | N/A | N/A |
| *Orobanche uniflora* | AY209297 | AF055144 | N/A | N/A | N/A |
| *Orobanche vallicola* | AY209295 | N/A | N/A | N/A | N/A |
| *Orthocarpus bracteosus* | KC480374 | JX023017 | KC542172 | AM233998 | KC542251 |
| *Orthocarpus tenuifolius* | KC480375 | JX023018 | JX091328 | AM234043 | KC542252 |
| *Parentucellia latifolia* | KC480376 | JX023019 | KC542173 | AM233999 | KC542253 |
| *Parentucellia viscosa* | KC480377 | JX023020 | AY849606 | AM234000 | KC542254 |
| *Pedicularis anthemifolia* | N/A | N/A | N/A | N/A | N/A |
| *Paulownia tomentosa* | N/A | AF055155 | AF051997 | AM234001 | KC542195 |
| *Pedicularis canadensis* | KC480378 | N/A | N/A | N/A | N/A |
| *Pedicularis confertiflora* | N/A | JX023021 | JX091329 | N/A | N/A |
| *Pedicularis contorta* | AY911245 | N/A | N/A | N/A | N/A |
| *Pedicularis cranolopha* | N/A | N/A | N/A | N/A | N/A |
| *Pedicularis densispica* | KC480379 | KC480430 | N/A | AM234002 | KC542255 |
| *Pedicularis elwesii* | AY949707 | JX023022 | AY949723 | N/A | KC542256 |
| *Pedicularis foliosa* | KC480380 | U48740 | AY949745 | AM234003 | N/A |
| *Pedicularis gyrorhyncha* | KC480381 | JX023023 | KC542174 | AM234004 | KC542257 |
| *Pedicularis ingens* | N/A | JX023024 | JX091330 | N/A | KC542258 |
| *Pedicularis julica* | KC480382 | JX023025 | KC542175 | AM234005 | KC542259 |
| *Pedicularis kansuensis* | KC480383 | N/A | N/A | AM234006 | N/A |
| *Pedicularis kerneri* | KC480384 | KC480431 | N/A | AM234007 | KC542260 |
| *Pedicularis lanceolata* | AY911247 | JX023026 | JX091331 | N/A | N/A |
| *Pedicularis rostratocapitata* | KC480385 | N/A | N/A | N/A | N/A |
| *Pedicularis sudetica* | AY911249 | N/A | N/A | N/A | N/A |
| *Pedicularis tuberosa* | KC480386 | JX023027 | KC542176 | AM234008 | KC542261 |
| *Phacellanthus tubiflorus* | KY706615 | KY706620 | KY706625 | KY706630 | KY706632 |
| *Phtheirospermum japonicum* | KC480387 | JX023028 | JX091332 | AM234010 | KC542262 |
| *Pterygiella nigrescens* | KC480388 | KC480432 | KC542177 | KC542192 | KC542263 |
| *Radamaea montana* | KC480389 | KC480433 | KC542178 | AM234044 | KC542264 |
| *Rehmannia glutinosa* | EU266023 | FJ172711 | GQ434277 | N/A | N/A |
| *Rhinanthus alectorolophus* | KC480390 | KC480434 | KC542179 | AM234012 | KC542265 |
| *Rhinanthus freynii* | KC480391 | KC480435 | KC542180 | AM234013 | KC542266 |
| *Rhinanthus glacialis* | KC480392 | KC480436 | KC542181 | AM234014 | KC542267 |
| *Rhinanthus minor* | KC480393 | JX023029 | KC542182 | AM234015 | KC542268 |
| *Rhynchocorys orientalis* | AY911251 | JX023030 | JX091333 | AM234016 | KC542269 |
| *Schwalbea americana* | KC480394 | JX023031 | AF051998 | AM234017 | KC542270 |
| *Scrophularia arguta* | N/A | JX023032 | JX091334 | AM234018 | N/A |
| *Sesamum indicum* | AF169853 | JN637766 | JN637766 | N/A | XM_011073075 |
| *Seymeria laciniata* | KC480395 | JX023033 | KC542183 | AM234019 | N/A |
| *Seymeria pectinata* | AY911253 | AF055141 | AF051999 | N/A | N/A |
| *Siphonostegia chinensis* | KC480396 | KC480437 | KC542184 | AM234020 | KC542271 |
| *Siphonostegia laeta* | KC480397 | N/A | N/A | N/A | KC542272 |
| *Siphonostegia syriaca* | KC480398 | N/A | N/A | N/A | N/A |
| *Solanum tuberosum* | LC020015 | DQ386163 | DQ386163 | DQ208423 | N/A |
| *Sopubia cana* | KC480399 | JX023034 | JX091335 | AM23402 | N/A |
| *Sopubia lanata* | KC480400 | KC480438 | KC542185 | AM23402 | KC542273 |
| *Sopubia ramosa* | KC480401 | N/A | N/A | AM23402 | N/A |
| *Striga asiatica* | KC480402 | U4874 | AF05200 | AM23402 | N/A |
| *Striga bilabiata* | KC480403 | JX023035 | JX091336 | AM23402 | KC542274 |
| *Striga elegans* | AY91125 | KC480439 | JX091337 | KC542193 | KC542276 |
| *Striga gesnerioides* | KC480404 | U4874 | AF48996 | AM23402 | KC542275 |
| *Striga linearifolia* | KC480405 | JX023036 | JX091338 | AM23402 | KC542277 |
| *Striga orobanchoides* | KC480406 | KC480440 | JX091339 | AM23402 | N/A |
| *Tozzia alpina* | KC480407 | U4875 | AF05200 | AM23403 | KC542278 |
| *Triphysaria floribunda* | KC480408 | N/A | N/A | N/A | N/A |
| *Triphysaria pusilla* | KC480409 | N/A | N/A | AM23403 | N/A |
| *Xylocalyx aculeolatus* | KC480410 | N/A | N/A | N/A | N/A |
| *Xylocalyx asper* | KC480411 | JX023037 | KC542186 | AM23403 | KC542279 |
| *Xylocalyx carterae* | KC480412 | N/A | N/A | AM23403 | N/A |
