# Supplementary material for: Testing the Hypothesis of Multiple Origins of Holoparasitism in Orobanchaceae: Phylogenetic Evidence from the Last Two Unplaced Holoparasitic Genera, Gleadovia and Phacellanthus
Source: Front Plant Sci. 2017 Aug 15;8:1380. doi: 10.3389/fpls.2017.01380 (PMC5559707; doi:10.3389/fpls.2017.01380)
Supplement: Table S4 — Summarized results of maximum likelihood analyses in Orobanchaceae based on single gene and combined matK and rps2 (plastid) data partitioned by gene. [file Table4.DOCX]

**Table S4.** Summarized results of maximum likelihood analyses in Orobanchaceae based on single gene and combined *mat*K and *rps2* (plastid) data partitioned by gene.

|  | ITS | *rps*2 | *mat*K | *rps*2 + *mat*K | *PHYA* | *PHYB* |
| --- | --- | --- | --- | --- | --- | --- |
| Genomes | nuclear | plastid | plastid | plastid | nuclear | nuclear |
| Species | 144 | 118 | 103 | 121 | 113 | 112 |
| Nucleotides after alignment | 739 | 710 | 1646 | 2555 | 1812 | 1944 |
| Parsimony-informative characters | 416 | 302 | 792 | 1090 | 1059 | 1028 |
| Clade I adjacent to root | + | **–** | + | + | **–** | + |
| Parasitic taxa form a clade | + | **–** | +, 82% | +, 94% | **–** | +, 95% |
| Clade I exists | +, 95% | **–** | +, 100% | +, 100% | +, 100% | +, 100% |
| Clade II exists | **–** | +, 98% | +, 100% | +, 100% | +, 76% | +, 100% |
| **Holoparasitic** **Clade III** exists | **–** | **–** | +, 85% | +, 75% | +, 99% | +, 76% |
| ***Gleadovia* is an independent lineage** | + | + | – | + | + | + |
| *Orobanche* form a clade | **–** | **–** | **–** | **–** | **–** | +, 88% |
| ***Phacellanthus tubiflorus* clusters with the species of section *Orobanche*** | +, 72% | +, 94% | +, 100% | +, 99% | +, 100% | +, 100% |
| Clade IV exists | +, 97% | **–** | +, 100% | +, 97% | +, 100% | +, 94% |
| Clade V exists | +, 100% | +, 53% | +, 100% | +, 98% | +, 100% | +, 100% |
| **The** **species of holoparasitic *Lathraea* form a clade** | +, 100% | +, 89% | +, 99% | +, 100% | **+**, 99% | +, 100% |
| Clade VI exists | +, 100% | **–** | +, 100% | +, 83% | +, 100% | +, 100% |
| **Holoparasitic** **species in Clade VI form a clade** | +, 70% | +, 50% | +, 100% | +, 68% | **–** | +, 100% |
| *Brandisia hancei* is an independent lineage | + | + | + | + | + | + |
| Three holoparasitic clade exists | **–** | **–** | + | + | **–** | + |

“+” indicates support (bootstrap values ≥ 50%), “**–**” means unsupported. Bootstrap values are indicated in each analysis which needs to be explained. All clades showed here follows McNeal *et al.* (2013): Clade I. *Lindenbergia*, Clade II. Cymbarieae, Clade III. Orobancheae, Clade IV. Pedicularideae, Clade V. Rhinantheae except *Pterygiella nigrescens*, and Clade VI. Buchnereae.
